# Modular (universal) CAR-T platforms *in vivo*: a comprehensive systematic review

**DOI:** 10.3389/fimmu.2024.1409665

**Published:** 2024-12-06

**Authors:** Afraa Mohammad, Anna Yurina, Tatiana Simonyan, Daniil Chistyakov, Rand Salman, Ksenia Zornikova, Elizaveta Minina, Apollinariya Bogolyubova

**Affiliations:** National Medical Research Center for Hematology, Moscow, Russia

**Keywords:** modular CAR T, universal CAR T, xenograft model, pre-clinical study, systematic review

## Abstract

**Background:**

Modular (universal) CAR T-platforms were developed to combat the limitations of traditional CAR-T therapy, allowing for multiple targeting of tumor-associated antigens and the ability to control CAR-T cell activity. The modular CAR-T platform consists of a universal receptor (signaling module) that recognizes an adapter molecule on the soluble module, which is responsible for antigen recognition. Multiple platforms have been developed over the last 12 years, and some of them have entered the clinical trial phase. This systematic review seeks to evaluate the different parameters of modular CAR-T platforms performance in animal models.

**Methods:**

A systematic search of literature in the PubMed database and in Google Scholar and BASE (Bielefeld Academic Search Engine) search engines was performed according to predefined eligibility criteria. All studies conducted on xenograft mouse models with any variant of modular CAR-T platforms were included. Forest plots were generated for visual presentation of the extracted quantitative findings (standardized mean difference (SMD) and median survival rate (MSR)).

**Results:**

A total of 33 studies employing 15 different modular CAR-T platforms were included. The platforms varied in terms of CAR-T cells, soluble module doses, and their frequency of administration. The studies showed a reduction in tumor burden and in tumor volume compared to the combined negative group. In comparison with the positive control group, there was no significant change in tumor burden or volume. In all the included studies the experimental group had a higher survival probability compared to the combined negative group at the study endpoint, with no significant difference in survival rate compared to the positive control group.

**Conclusion:**

The modular CAR-T platforms are generally effective and are a valuable addition to the arsenal of CAR therapy.

**Systematic Review Registration:**

https://www.crd.york.ac.uk/prospero/ PROSPERO, identifier CRD42023443984.

## 1 Introduction

**Figure 1:**
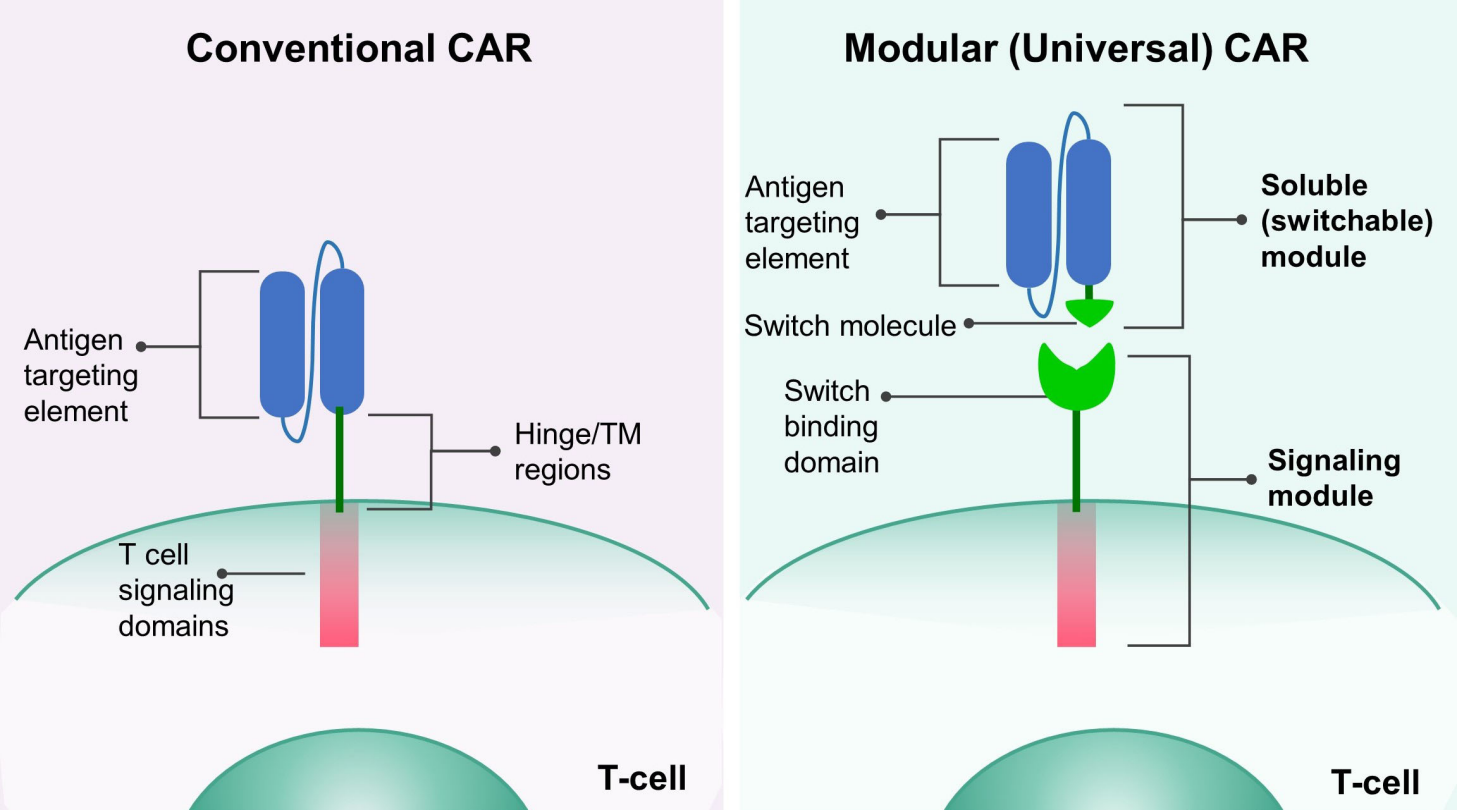
The design of modular CAR platform. The conventional CAR **(left)** consists of an antigen-targeting element, a hinge and transmembrane (TM) domain, and an intracellular signaling domain. In the modular (universal) CAR **(right)**, the antigen targeting element and the hinge/TM domain are separated, and a switch molecule is attached to the antigen targeting element, forming the soluble (switchable) module. The signaling module has a switch binding domain, which enables the interaction between the signaling and the soluble module.

The development of chimeric antigen receptor (CAR) T-cell therapy marked a novel era for personalized cancer treatment. Increasing evidence from clinical trials suggests that CAR T therapy will continue to be a potential new standard of care for second-line treatment of B-cell lymphoma (1, 2). However, such success was not observed in treating solid tumors, which can be attributed to multiple factors, including the immunosuppressive tumor microenvironment, the inability of CAR-T cells to migrate and infiltrate tumors, and loss of antigen expression (3). In addition, various reports have shown clinical evidence of CAR-T-associated toxicities linked to the uncontrolled activity of CAR-T cells, on-target/off-tumor effects, and off-target toxicities (4, 5). Hence, advancements in CAR-T cell design have become necessary to tackle these obstacles and reduce safety risks. One such advancement is the modular CAR T technology, which uses a switch molecule to separate T cell signaling domains and targeting elements, enabling precise control over CAR T cells. Unlike traditional CAR T cells targeted directly at the tumor antigen, modular CAR T cells target an adapter or switch element. This adapter is connected to the tumor antigen-binding domain, serving as a bridge between CAR and the tumor antigen. Consequently, this platform consists of two components: a signaling module, comprising a universal CAR expressed on T-cell surfaces, and a soluble or switchable module that links the CAR to tumor-associated antigens (**Figure 1**). Modular CAR T technology enables the simultaneous targeting of numerous antigens using multiple soluble modules, eliminating the necessity for extensive re-engineering of CAR. These features are seen as advantageous for combating relapse, mitigating over-activation, and improving specificity (6, 7). The development of modular CAR-T platforms commenced approximately 12 years ago, and various efforts have been made to bring them into clinical phase. The initial trials tested CD16-CAR in combination with rituximab, SEA-BCMA, and trastuzumab for patients with B-cell lymphoma, multiple myeloma, and HER2 positive solid tumors, respectively (ClinicalTrials.gov ID: NCT02776813; NCT03266692; NCT03680560). However, the first two trials were terminated due to CAR-associated toxicities. Since 2020, six clinical trials have been initiated to examine the effectiveness of the UniCAR, the sCAR, the anti-FITC CAR, and the CAR-CD19 platforms. These trials are currently in the process of recruiting participants and have yet to produce any primary results (ClinicalTrials.gov ID: NCT04633148; NCT04230265; NCT04450069; NCT04488354; NCT05312411; NCT06045910). Additionally, two clinical trials using the ARC-SparX platform are currently recruiting patients with relapsed and refractory multiple myeloma (NCT04155749) and patients with relapsed and refractory acute myelogenous leukemia or high-risk myelodysplastic syndrome (NCT05457010).

The evaluation of the efficacy and safety of modular CAR-T platforms is performed mainly on xenograft models of immunodeficient mice, most commonly NSG mice. These models aid in assessments of the trafficking and proliferation profiles and the anti-tumor activity of CAR T cells; however, issues like cross-reactivity and difficulty predicting human responses persist (8, 9). There is an ongoing debate about whether the animal models are appropriate for CAR-T therapy pre-clinical assessment, and regulations about animal use are periodically updated based on the available evidence. In this regard, systematic reviews (SR) and meta analyses offer a comprehensive and transparent overview of available information (10).

Multiple modular CAR-T platforms currently exist, as summarized in **Table 1**. While these platforms share a common design principle, their performance can vary significantly due to differences in the components of each platform and their properties. Consequently, determining which platform holds the most promise for clinical translation is unfair. However, by systematically identifying, appraising, and synthesizing all available research evidence, our study aims to provide a transparent overview of *in vivo* studies of modular CAR-T platforms. This approach will enhance our understanding of their performance in animal models, highlighting the advantages and disadvantages of these innovative platforms.

**Table 1:** Modular CAR-T platforms.

Modular CAR platform name	Switch element	Switch-binding domain	Reference
Biotin-binding immunoreceptor (BBIR)	Biotin	Avidin	(55)
Anti-FITC CAR	Fluorescein isothiocyanate (FITC)	Anti-FITC scFv	(53)
SpyTag-SpyCatcher Universal CAR	SpyTag	SpyCatcher	(54)
split, universal, and programmable (SUPRA) CAR	Leucine zipper	Leucine zipper	(22)
ConvertibleCAR	NKG2D ligand U2S3	Mutant NKG2D	(35)
switchable CAR-T cells (sCAR)	Peptide neo-epitope (PNE)	Anti-PNE scFv	(24)
UniCAR	5B9 tag	Anti-5B9 tag scFV	(26)
RevCAR	Anti-5B9 tag scFV	5B9 tag	(63)
SNAP CAR	Benzylguanine	SNAPtag	(23)
CAR-CD19	CD19 ECD	Anti-CD19 scFv	(41)
Barstar-based CAR (BsCAR)	Barnase	Barstar	(32)
CD16 CAR	Ab Fc region	CD16 ECD	(38)
Fabrack-CAR	Meditope peptide	Fab arm of mAbs	(28)
Target-redirected universal CAR (TRUE)	F-AgNP	Anti-EGFRvIII scFV	(34)
Synthetic agonistic receptor (SAR)	Anti-EGFRvIII scFv	EGFRvIII	(49)
Fc-targeting CAR	P329G L234A/L235A (LALA)	Anti-P329G LA/LA scFv	(48)
Latching Orthogonal Cage–Key pRotein (LOCKR) system	‘Cage’ protein that contains ‘Latch’ sequestered peptide Bim	Bcl2 binder	(57)
Adapter CAR (AdCAR) system	Linker and label (Biotin)	scFV mBio3	(58)
ARC-SparX platform	TAG domain (AFP domain III)	Tag-binding D-Domain	(59)
Conduit CAR	GLPB30 anti-(G4S)_n_ linker antibody	Linker sequence in scFv	(60)
Folate receptor (BsAb-binding immune receptor (BsAb-IR))	Folate	Extracellular domain of folate receptor	(61)

## 2 Material and methods

### 2.1 Literature search

The study protocol was agreed upon and registered in PROSPERO (CRD42023443984) on July 18, 2023. This report was prepared in accordance with the PRISMA guidelines and checklist (11).

#### 2.1.1 Eligibility criteria

The eligibility criteria were predefined within the study protocol, aligning with each aspect of the PICOS framework (Population/Animals, Intervention, Comparator(s)/Control, Outcome Measure(s), and Study design):

1. Population/Animals: Xenograft mouse models were included, encompassing both cell-line derived and patient-derived models, while excluding other species such as humans, zebrafish, dogs, and non-human primates, as well those for non-cancerous diseases.
2. Intervention: All variants of the modular CAR-T platform *in vivo* studies, with non-human CAR T interventions being excluded.
3. Comparators: Various control cohorts were considered, including untreated mice, mice treated with conventional CAR-T cells or only CAR-T cells without the switchable module, non-transduced T cells, or only the switchable module, with noncomparative studies being excluded, alongside those reporting normalized data.
4. Outcome measure(s): Primary outcomes included tumor burden, tumor volume, and median survival, while secondary outcomes comprised percent tumor-free, peripheral blood T cell quantification post-T-cell injection, peripheral blood T cell phenotype, human cytokines in the peripheral blood, body weight, metastases formation, and pharmacokinetic properties of the soluble module.
5. Study design: All English-language, full-text, controlled interventional animal studies were eligible for inclusion, while excluding review articles, noncomparative studies, commentaries, editorials, case reports, case series, and other study types.

#### 2.1.2 Search strategy

To collect fitting studies, we conducted comprehensive research in the PubMed database and in Google Scholar and BASE (Bielefeld Academic Search Engine) search engines. The search strategy was developed in accordance with the step-by-step guide of Leenaars et al. (12). The full search strategy is available in the PROSPERO protocol and in the **Supplementary Materials**.

#### 2.1.3 Study selection

All identified records were imported into EndNote (RRID: SCR_014001) for the management of search records. Following the removal of duplicates, two reviewers (AM and EM) independently screened the titles and abstracts of the studies. Subsequently, two researchers (KZ and EM) independently reviewed the full-text articles for inclusion. In instances of disagreement, consensus on inclusion or exclusion was achieved through discussion, with involvement from a third researcher (AM) if necessary.

### 2.2 Data extraction

We designed a data extraction form using Google Forms (RRID: SCR_023174), comprising three sections: open-ended questions (e.g., year of publication, country of origin, definition of control groups, CAR-T/tumor cell dosage, etc.), closed-ended questions (e.g., mice gender, type of xenograft model, reports of dropouts, etc.), and an upload form for quantitative data as an Excel spreadsheet. The form underwent pilot testing and was then used for data collection. The included studies were randomly assigned to four reviewers (AY, TS, DC, and RS). Each paper was independently reviewed by at least two reviewers to ensure data collection accuracy. Extracted data were compared, and any discrepancies were resolved through discussion. Data related to study characteristics, animal model, intervention details, and primary and secondary outcome measures were extracted. Details on the data to be extracted are available in the PROSPERO protocol. In cases where raw data were not presented, graphical data were digitized using WebPlotDigitizer (version 4.7., RRID: SCR_013996).

### 2.3 Risk of bias assessment

We evaluated the risk of bias in the included studies using the Systematic Review Centre for Laboratory Animal Experimentation (SYRCLE) risk of bias tool for animal intervention studies (13). Specifically, we addressed the following domains: 1) selection bias, which included assessment of sequence generation, baseline characteristics, and allocation concealment; 2) performance bias, examining random housing and blinding of investigators; 3) detection bias, considering random outcome assessment and blinding of assessors; 4) attrition bias, focusing on incomplete outcome data; 5) reporting bias, assessing selective outcome reporting.

Additionally, we investigated other potential sources of bias, such as contamination due to dietary influences on outcomes, unit of analysis errors, and design-specific risk of bias where treatment was introduced prior to or concurrently with tumor cells. Two reviewers (AM and EM) independently applied the tool to each study, documenting supporting information and justifications for the risk of bias judgments (classified as low, high, or unclear) in each domain. Any discrepancies in judgments or justifications were resolved through discussion, with a third author (AB) serving as an arbiter if necessary.

### 2.4 Data processing

For continuous outcomes such as tumor burden, tumor volume, body weight, and human cytokine levels, we computed standardized mean difference (SMD) effect sizes (Hedge’s G) along with their corresponding 95% confidence intervals (CIs). Given that the included studies utilized diverse measurement scales to evaluate these outcomes, standardization was necessary to facilitate comparison (14).

Survival data were handled differently, with the median survival values derived from published Kaplan-Meier curves by drawing a horizontal line at 50% on the y-axis and determining its intercept with the curve. In cases where the curve was horizontal at y = 50%, the average of the first and last time points of the line was considered the median. Studies where the median survival could not be calculated at the experiment’s endpoint were not excluded. In such instances, the median survival was considered the last assessment point, with more than 50% of the animals surviving at this time (14, 15). Effect sizes for individual studies were computed by taking the logarithm of the quotient of the median survival in the experimental group by that in the control group (median survival rate (MSR)). The precision of survival studies was weighted based on the number of animals included.

In cases where multiple control or experimental groups were present, relevant experimental groups were combined into a single group, as were relevant control groups (16).

### 2.5 Data synthesis

Meta-analysis was not conducted due to the variability in interventions and experimental settings. However, forest plots were generated for visual comparisons with combined negative or positive groups. In cases where multiple targets or tumor models were utilized, outcomes for each were analyzed separately alongside their corresponding controls, with each study being identified with a specific identifier. In addition, a summary table was used to summarize outcome data, or a descriptive summary was provided. Between-study heterogeneity variance was assessed using *I^2^* statistics, along with its 95% confidence intervals. An *I^2^* threshold of >75% was arbitrarily adopted to indicate considerable heterogeneity, with additional consideration given to the evidence for this heterogeneity and its visualization on forest plots. To explore patterns of heterogeneity, Graphic Display of Heterogeneity (GOSH) plots were utilized, and sensitivity analyses were conducted by removing outlier studies. Funnel plots and Egger’s test for asymmetry to detect publication bias were not performed, as suggested by the Grading of Recommendations, Assessment, Development, and Evaluation (GRADE) approach, which advises considering downgrading for publication bias in studies with predominantly small sample sizes, positive results, and commercial funding (17). All analyses were conducted in the software environment R (R Project for Statistical Computing (RRID: SCR_001905)) using the meta (RRID: SCR_019055), metafor (RRID: SCR_003450), and dmetar (RRID: SCR_019054) packages according to the hands-on guide (18).

## 3 Results

### 3.1 Study selection

**Figure 2:**
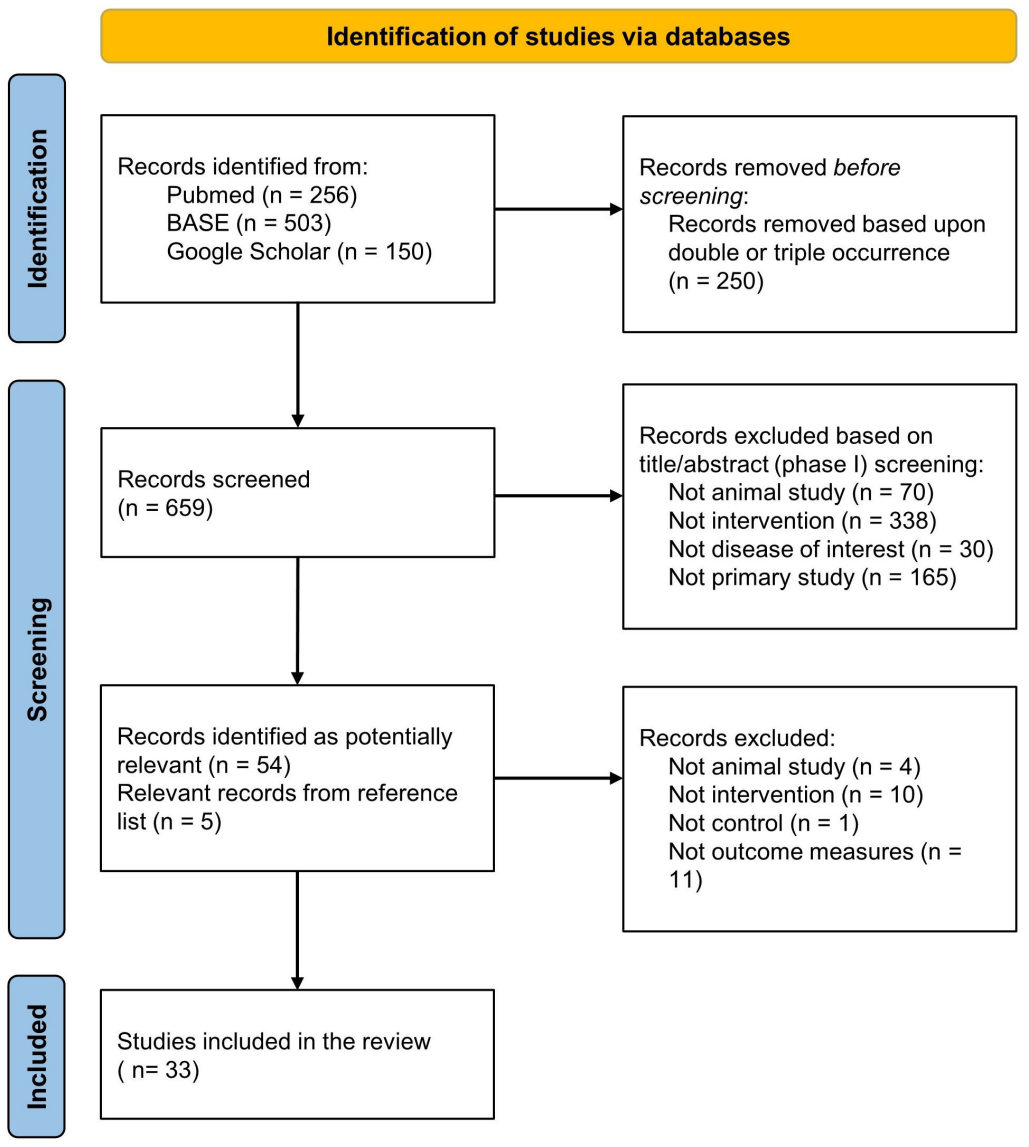
Preferred Reporting Items for Systematic Reviews and Meta-analysis (PRISMA) flow diagram for the included studies.

A comprehensive systematic literature search yielded a total of 909 studies. Following the removal of duplicate entries, 659 studies underwent an eligibility assessment through title/abstract screening, of which 54 were identified as potentially relevant and their reference lists were screened for eligible records. As a result, 5 more studies were added to the pool of studies that underwent a full-text screening process. 26 studies were excluded based on predetermined criteria: Four reports were excluded as they did not involve animal experimentation. Ten reports were excluded because they did not utilize a modular CAR T platform. One report was excluded due to the absence of appropriate controls. One report was excluded as it did not encompass any of the specified outcome measures outlined in the inclusion criteria. Nine reports were excluded since they reported tumor burden data for a duration of less than 7 days. The study selection process is presented in **Figure 2**. Ultimately, a total of 33 studies met the eligibility criteria and proceeded to undergo data extraction and subsequent analysis.

### 3.2 Characteristics of animal models in included studies

#### 3.2.1 Baseline characteristics

**Figure 3:**
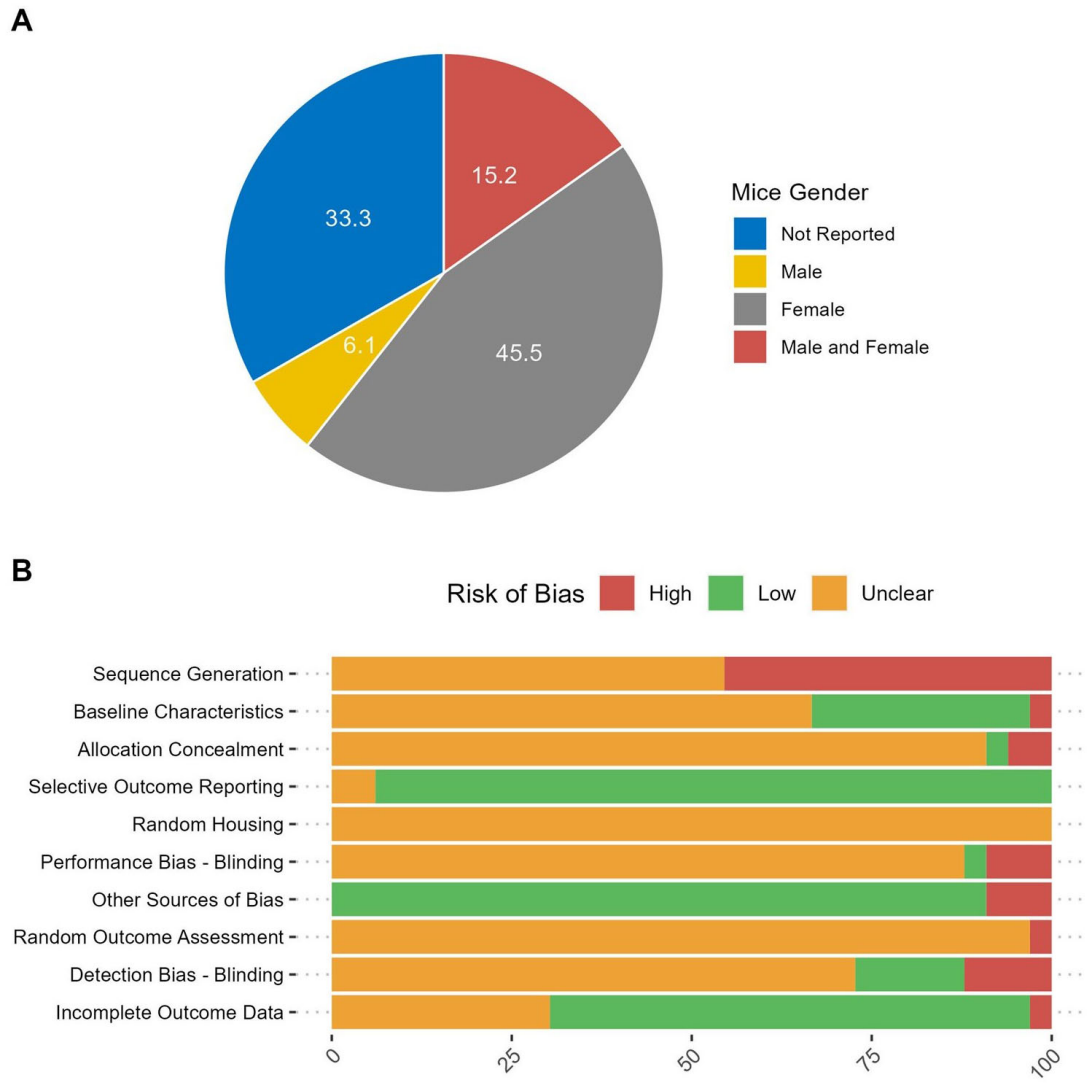
Study characteristics. **(A)** The percent of included studies reporting the gender of mice used in the experiments. **(B)** Risk of bias in the included studies.

One or more of the baseline characteristics of the murine population (strain, age, sex, total number, and provider of the mice) were adequately reported in the majority of studies. Notably, 42% (14 out of 33) of the included studies provided comprehensive details across all five categories. The ages of the mice varied between studies, with the highest range being 6 to 20 weeks and a median age of 7 weeks. In 45% (15 out of 33) of the studies, exclusively female mice were used. In contrast, only 6% (2 out of 33) of studies employed male mice. 15% (5 out of 33) of experiments employed both male and female mice, whereas the remaining 33% (11 out of 33) studies did not disclose this information. (**Figure 3A**). The total number of mice was either provided directly or deduced from the experimental data. Otherwise, if the number could not be calculated, it was classified as ‘not reported,’ which accounted for 18% (6 of 33) of the studies. The overall number of mice enrolled in all the experiments was 1595, with a median of 50 per study.

#### 3.2.2 Types of *in vivo* models

Most of the included studies (28 out of 33) employed the NSG mouse model (NOD.Cg-*Prkdc^scid^Il2rg^tm1Wjl^/*SzJ), while one study used the NOG mouse model (NOD.Cg*-Prkdc^scid^Il2rg^tm1Sug^/*ShiJic) and one study used the NXG mouse model (NOD*-Prkdc^scid^Il2rg^Tm1^/*Rj). These models are characterized by the absence of functional T and B lymphocytes and exhibit multifaceted defects in NK cell activity, macrophage function, complement activity, and dendritic cell function. Despite distinct *Il2rg* targeted mutations and different NOD backgrounds, these models are considered equivalent regarding overall biological/physiological characteristics and experimental applications (19). Additionally, two studies utilized athymic nude mice carrying the “nude” mutation (*Foxn1^nu^*), which results in defects in T cell development. One study used nude mice on a BALB/C background (BALB/c Nude), while the other on a NMRI background (NMRI-*Foxn1^nu^/Foxn1^nu^*).

The study by Pennell et al. employed a synergic mouse model on a C57BL/6 background where mice B-cells exclusively express human CD19 (huCD19^Tg/0^). These mice were then implanted with a mouse lymphoma cell line engineered to co-express human CD19 (TBL12.huCD19) and received syngeneic murine T cells expressing a human CD19-directed CAR. This experimental design aimed to replicate clinical cytokine release syndrome (CRS) and neurotoxicity.

#### 3.2.3 Tumor establishment

Tumors can be established using either human tumor cell lines (cell line-derived tumor xenografts, CDX) or tumor samples from patients (patient-derived xenografts, PDX). For models of hematopoietic-origin tumors cells are typically injected systemically via intravenous (IV) route. Solid tumors can be established by subcutaneous (SC) or intraperitoneal (IP) inoculation. To create a more realistic tumor environment, orthotopic inoculation (OI) is used, while systemic inoculation via IV route is employed to mimic tumor invasion and metastasis.

Patient-derived xenografts (PDXs) generated from clinical tumor samples are valuable models that closely replicate the genetic and cellular profiles of the original tumors. Among the included studies, Loff et al. and Raj et al. utilized PDXs to study the anti-tumor activity of the UniCAR and switchable CAR (sCAR) platforms, respectively. Loff et al. developed a CD123-expressing B-ALL xenograft model to evaluate the anti-tumor activity of UniCAR-T cells against extramedullary leukemic bulks by subcutaneously transplanting tumor cells into the flanks of NSG mice (20). On the other hand, Raj et al. employed an orthotopic model of advanced pancreatic ductal adenocarcinoma (PDAC) by inoculating stage IV PDAC cells into the pancreas of immunocompromised NSG mice (21). These cells express HER2 at relatively modest levels, creating stringent conditions for CAR-T therapy. This model establishes highly aggressive primary tumors with rapid-onset metastasis to the liver and lungs, mimicking late-stage PDAC in patients.

Most of the included studies (31 out of 33) established CDX models using popular cell lines that express the target antigen(s). For solid tumor establishment, the majority introduced the tumor antigen via the subcutaneous (SC) route (14 out of 33) or the intraperitoneal (IP) route (4 out of 33). Additionally, 8 studies utilized the intravenous (IV) route to establish hematopoietic-origin tumor models, with the exceptions of Cho et al. and Ruffo et al., which used human epidermal growth factor receptor 2 (HER2)-expressing cell lines to study the activity of CAR-T cells against metastatic tumor cells (22, 23).

The included studies targeted a range of hematologic and solid tumor antigens. Hematologic tumor antigens included CD33 (3 out of 33 studies), CD123 (3 out of 33 studies), CD20 (5 out of 33 studies), and CD19 (3 out of 33 studies). For solid tumors, the targeted antigens were HER2 (9 out of 33 studies), prostate stem cell antigen (PSCA) (2 out of 33 studies), receptor tyrosine kinase AXL (1 out of 33 studies), mesothelin (3 out of 33 studies), B7-H3 (CD276) (1 out of 33 studies), epidermal growth factor (EGFR) (4 out of 33 studies), cadherin-6 (CDH6) (1 out of 33 studies), folate receptor (FR) (2 out of 33 studies), carbonic anhydrase IX (CAIX) (1 out of 33 studies), prostate specific membrane antigen (PSMA) (2 out of 33 studies), glypican-3 (GPC3) (1 out of 33 studies), ROR1 (1 out of 33 studies), C-type lectin domain family 12 member A (CELC12A) (1 out of 33 studies), disialoganglioside (GD2) (1 out of 33 studies), and epithelial cell adhesion molecule (EpCAM) (1 out of 33 studies).

Discussion regarding the immunogenicity of CAR-T platform components was addressed in a relatively modest proportion of studies, with only 36% (12 out of 33) acknowledging or examining this aspect. Rodgers et al. specifically investigated the immunogenicity risk associated with engrafting a nonhuman sequence into an antibody fragment (Fab) to create a switch. They conducted an *in silico* immunogenicity analysis of the adapter linked to the N terminus of the light or heavy chains of a model, therapeutically approved antibody (trastuzumab). This analysis predicted that the PNE graft had a low likelihood of inducing an antibody response in the context of a typical antibody (24).

Regarding euthanization criteria, 54% (18 out of 33) of the studies presented explicit criteria, while merely 12% (4 out of 33) included comprehensive assessments of overall body condition and coat condition. Furthermore, a subset of studies, constituting 15% (5 out of 33), employed a secondary mouse strain for various specific purposes, including pharmacokinetics, T-cell depletion, pharmacokinetics-half life, pharmacokinetics-biodistribution, and breeding of the huCD19^Tg/0^ strain. Detailed characteristics of the animal models are described in **Table 2**.

**Table 2:** The characteristics of the included studies.

Author, year	CAR Name ^a^	Soluble module ^b^	Target antigen(s) ^c^	Mouse strain ^d^	Gender of mice	Age of mice (weeks)	Xenograft model (No. of cells, Route of administration) ^e^	Ref
Ambrose et al., 2021	CAR-CD19	scFv—CD19 ECD	HER2	NSG	Not reported	Not reported	1×10^6^ SKOV3, SC	(41)
Bejestani et al., 2017	UniCAR	scFV—La/SS-B 5B9	PSCA	NSG	Male	5-8	1×10^6^ PC3-PSCA, SC	(36)
Benmebarek et al., 2021	SAR	scFV—scFV (tnFv)	CD33	NSG	Female	4	1×10^6^ THP-1, IV 2×10^6^ MV4-11, IV	(52)
Cartellieri et al., 2016	UniCAR	scFV—La/SS-B 5B9	CD33, CD123	NSG	Not reported	8-10	0.5×10^6^ MOLM-13, IV	(26)
Cho et al., 2018	SUPRA CAR	scFV—Leucine zipper	HER2, Axl, Mesothelin	NSG	Female	4-6	7.5×10^6^ SK-BR-3, IP 5×10^6^ Jurkat T, IV	(22)
He et al., 2021	sCAR	Nb—PNE	CD13	NSG	Not reported	8-12	10×10^6^ THP-10, SC	(30)
Hidalgo et al., 2023	Anti-FITC CAR	Ab—FITC	B7-H3	NSG	Not reported	8-12	1×10^6^ 143B, SC	(64)
Karches et al., 2019	SAR	BiAb	Mesothelin	NSG	Not reported	Not reported	0.5×10^6^ Suit-2-MSLN, SC 0.5×10^6^ MIA PaCa-MSLN, SC 1×10^6^ MSTO-MSLN, SC	(49)
Kegler et al., 2019	UniCAR	scFV—La 5B9	PSCA	NMRI-Foxn1^nu^/Foxn1^nu^	Male	8	1×10^6^ PC3-PSCA, SC	(45)
Kudo et al., 2014	CD16 CAR	mAb	CD20, HER2	NSG	Not reported	Not reported	0.3×10^6^ Daudi, IP	(47)
Kuo et al., 2021	Fabrack-CAR	memAb	EGFR/HER3, CDH6	NSG	Female	Not reported	5×10^6^ OVCAR3, IP	(28)
Landgraf et al., 2020	Convertible CAR	MicAbody™	CD20	NSG	Female	6	1×10^6^ Raji, SC 0.5×10^6^ Raji, IV	(35)
Lee et al., 2018	Anti-FITC CAR	Ligand—FITC	FR, PSMA, CA IX	NSG	Not reported	Not reported	2×10^6^ MDA-MB-231, SC	(44)
Lee et al., 2019	Anti-FITC CAR	Ligand—FITC	PSMA	NSG	Not reported	Not reported	MDA-MB-231, SC	(43)
Liu et al., 2020	Spy-Catcher CAR	scFv—SpyTag	GPC3	NSG	Female	6-8	5×10^6^ HepG2, SC	(51)
Loff et al., 2020	UniCAR	scFV—La/SS-B 5B9	CD123	NSG	Male and Female	8-12	0.5×10^6^ MOLM-13, IV 1×10^6^ PDX B-ALL, SC	(20)
Lu et al., 2019	Anti-FITC CAR	Ligand—FITC	FR	NSG	Female	4-5	2.5×10^6^ MDA-MB-231, SC 5×10^6^ THP1-FRb, SC 1×10^6^ -FRa, SC	(50)
Ma et al., 2015	Anti-FITC CAR	Fab—FITC	CD19	NSG	Female	6-8	0.5×10^6^ Nalm-6, IV	(29)
Meyer et al., 2021	UniCAR	scFV—La/SS-B 5B9	CD123	NSG	Male and Female	8-12	0.5×10^6^ MOLM-13, IV	(37)
Minutolo et al., 2020	Spy-Catcher CAR	mAb—SpyTag	HER2	NSG	Female	6-12	1×10^6^ SKOV3-CBG+GFP, IP	(54)
Ochi et al., 2014	CD16 CAR	mAb	CD20	NOG	Female	6	0.5×10^6^ Raji, IV	(38)
Peng et al., 2022	sCAR	Fab—PNE	ROR1	NSG	Male and Female	6-8	0.5×10^6^ JeKo-1, IP 0.5×10^6^ HT-29, IP	(39)
Pennell et al., 2022	sCAR	scFv—PNE	CD19	huCD19^Tg/0^	Not reported	8-16	1×10^6^ TBL12.huCD19s, IP	(65)
Raj et al., 2018	sCAR	Fab—PNE	HER2	NSG	Not reported	6-8	0.1×10^6^ PDX, OI	(21)
Rennert et al., 2021	CAR-CD19	scFV/Nb—CD19 ECD	CD33, CLEC12A	NSG	Not reported	6-8	1×10^6^ Nalm6, IV 0.1×10^6^ U937, IV 1×10^6^ PL21, IV	(42)
Rodgers et al., 2015	sCAR	Fab—PNE	CD19	NSG	Female	9-11	0.5×10^6^ Nalm-6, IV	(24)
Ruffo et al., 2023	SNAP CAR	mAb—SNAP tag	HER2	NSG	Female	4-6	0.5×10^6^ Nalm-6, IV	(23)
Saleh et al., 2023	RevCAR	scFv—scFv E7B6 or E5B9	EGFR, GD2	NXG	Female	8	1×10^6^ U251, SC	(46)
Stepanov et al., 2022	BsCAR	DARPins—barnase	HER2, EpCAM	NSG	Male and Female	6-8	2×10^6^ BT-474, SC	(32)
Stock et al., 2022	Fc-targeting CAR	P329G L234A/L235A (LALA)	Mesothelin, HER2	NSG	Female	8-20	1×10^6^ MSTO-MSLN, SC 1×10^6^ HCC1569-HER2, SC	(48)
Su et al., 2022	CAR-CD19	scFv—CD19 ECD	CD20	NSG	Female	6-10	2.5×10^6^ JeKo-CD19, IV	(40)
Sun et al., 2022	TRUE CAR	F-AgNPs	EGFR vIII	BALB/c Nude	Female	5	5×10^6^ MGC803, IP 3×10^6^ MKN45, SC	(34)
Tamada et al., 2012	Anti-FITC CAR	mAb—FITC	EGFR, HER2, CD20	NSG	Male and Female	6-10	1-2×10^6^ SW480, SC 1-2×10^6^ AU565, SC 1-2×10^6^ Panc 6.039, SC	(53)

a SAR, Synthetic agonistic receptor; SUPRA, A split; universal; and programmable CAR system; sCAR, switchable CAR; BsCAR, Barstar-based CAR; TRUE, Target-redirected universal CAR-T.

b Soluble module is represented as Ag-binding domain—CAR-binding domain; scFv, single chain variable fragment; Nb, Nanobody; Ab, Antibody; mAb, monoclonal Antibody; memAb, meditope-enabled mAb; MicAbody™, Bispecific adapter comprised of an iNKG2D-exclusive ULBP2-based ligand fused to an antigen-targeting antibody; F-AgNPs, Fusogenic antigen loaded nanoparticles.

c HER2, Human epidermal growth factor receptor 2; PSCA, Prostate stem cell antigen; AXL, AXL receptor tyrosine kinase; EGFR, epidermal growth factor; CDH6, Cadherin-6; FR, folate receptor; PSMA, prostate specific membrane antigen; CA IX, Carbonic anhydrase IX; GPC3, Glypican-3; CLEC12A, C-type lectin domain family 12 member A; GD2, disialoganglioside; EpCAM, Epithelial cell adhesion molecule.

d NSG, NOD.Cg-*Prkdc^scid^ Il2rg^tm1Wjl^*/SzJ or NOD SCID gamma; NOD SCID, immunodeficient nonobese diabetic/severe combined immunodeficiency; NOG, NOD.Cg-*Prkdc^scid^ Il2rg^tm1Sug^*/ShiJic; NXG, NOD-*Prkdc^scid^ Il2rg^tm1^*/Rj.

e PDX, patient derived xenograft; SC, subcutaneous; IV, intravenous; IP, intraperitoneal; OI, orthotopic inoculation.

### 3.3 Characteristics of intervention in included studies

#### 3.3.1 Platform design in the included studies

The modular CAR platforms varied across studies depending on the adapter molecules used to facilitate the interaction between the soluble and signaling modules. Among the included studies, 18% (6 out of 33) utilized the anti-FITC CAR platform, wherein the universal CAR recognizes antibodies or ligands tagged with fluorescein isothiocyanate (FITC) molecules. Since FITC was shown to be safe for in-human use in conjugation with monoclinal antibodies for tumor-specific fluorescence imaging (25), it has the potential for use in cellular immunotherapy. However, the short half-life of the soluble module necessitates multiple injections at short intervals.

Another 15% (5 out of 33) of the studies adopted the UniCAR platform, developed by Michael Bachmann’s laboratory at Helmholtz-Zentrum Dresden-Rossendorf (HZDR) in Germany. This platform employs a 10-amino acid sequence derived from the nuclear protein La/SS-B (5B9 tag) as the adapter molecule. The primary goal of this design is to provide rapid and reversible control of CAR T cell activity, enable multiple targeting, and reduce the risk of developing antigen-free tumors during treatment. UniCAR contains humanized anti-La 5B9 scFv as the ectodomain of CAR, allowing CAR T cells to target multiple antigens either sequentially or concurrently. While this model offers significant flexibility, potential immunogenicity is a concern. The La protein is a known autoantigen in Sjögren’s syndrome and systemic lupus erythematosus, and autoantibodies against La epitopes are common in patients and may also exist in healthy populations (26).

Additionally, 15% (5 out of 33) of the studies employed the switchableCAR platform (sCAR), which features a 14-amino acid peptide neo-epitope (PNE) sequence from the GCN4 yeast transcription factor as the adapter. This platform is advantageous because the PNE sequence does not exist in the human proteome, has a high-affinity antibody, and has been found to be non-immunogenic. The anti-PNE CAR utilizes the PNE-antibody (52SR4) scFv as the ectodomain. However, despite the non-existence of the PNE sequence in the human proteome, antigenic mimicry may still result in off-target activation of CAR T-cells.

9% (3 out of 33) of the studies employed the conventional anti-CD19 CAR, equipped with a CD19-containing bridging protein (BP) capable of redirecting CAR19 T cells towards a range of target antigens. The BP comprises the extracellular domain of human CD19 coupled with disulfide-stabilized single-chain Fv (scFv) or nanobodies (Nb) targeting the respective antigen.

Furthermore, the following CAR platforms were each represented by two studies:

##### 3.3.1.1 Spy-catcher CAR

This platform utilizes the SpyTag/SpyCatcher protein ligation system, featuring the extracellular enzyme SpyCatcher (116 amino acids), which forms a spontaneous amide bond with SpyTag found in the soluble module. The SpyTag/SpyCatcher system boasts high affinity between its components, allowing the soluble module to be used at much lower concentrations. This can reduce treatment-related side effects and lower treatment costs. However, both the SpyTag and SpyCatcher sequences are derived from *Streptococcus pyogenes*, which may have the potential to induce an immune response and reduce antitumor effects.

##### 3.3.1.2 Synthetic Agonistic Receptor (SAR)

SAR is composed of an extracellular domain derived from human EGFRvIII, which binds to an anti-hEGFRvIII scFv conjugated with an antigen-specific scFv to create a bispecific antibody (BiAb).

##### 3.3.1.3 CD16 CAR

The CD16 CAR platform is based on CD16 (FcγR IIIa) antibody receptors present in various immune cells. CAR T-cell activity in this case is mediated through antibody-dependent cell-mediated cytotoxicity (ADCC). CD16 CAR employs the high-affinity CD16 V158 variant for its extracellular domain, which binds to the Fc part of antibodies. Despite that this system allows for the use of clinically available tumor-targeting antibodies as the soluble module, the major disadvantage is the potential autoimmune reactivity. The authors proposed a way to solve this issue by the transient expression of CAR using mRNA electroporation. However, this strategy has yet to be studied.

Finally, the following CAR platforms were each represented by one study:

##### 3.3.1.4 Split, universal, and programmable CAR system (SUPRA CAR)

SUPRA CAR uses the high-affinity heterodimeric interaction between basic leucine zipper pairs, a main component of many transcription factors. These peptide domains are naturally present in cells and are not immunogenic. This system is advantageous in terms of its ability to control CAR T cell activity and phenotype by adjusting multiple variables (1): the affinity between leucine zipper pairs (2), the affinity between tumor antigen and scFv (3), the concentration of zipFv, and (4) the expression level of zipCAR. Additionally, SUPRA CAR can be used to logically control the activity of CAR T cells.

##### 3.3.1.5 RevCAR

The RevCAR T platform is an inverse version of the UniCAR platform, with the 5B9 tag serving as the extracellular domain of CAR.

##### 3.3.1.6 Fc-targeting CAR

The Fc-targeting CAR platform is designed to target the P329G L234A/L235A (LALA) mutation found in the Fc region of therapeutic tumor-targeting human antibodies.

##### 3.3.1.7 Fabrack-CAR

Fabrack-CAR has a cyclic, twelve-residue meditope peptide in the extracellular domain. This meditope binds to meditope-enabled monoclonal antibodies (memAbs) via an engineered binding pocket within the Fab arm.

##### 3.3.1.8 ConvertibleCAR

This platform contains an inert form of the human NKG2D extracellular domain (iNKG2D) that recognizes an iNKG2D-specific ULBP2-S3 variant-based ligand fused to an antigen-targeting antibody (MicAbody). The innovative application of this platform is the delivery of cytokines selectively to iNKG2D-CAR expressing cells which has the potential to not only promote their expansion, but also to use differential cytokine signaling to control T cell phenotype and function.

##### 3.3.1.9 BsCAR (Barstar-based CAR)

The BsCAR platform makes use of the extraordinary affinity of the barnase-barstar toxin-antitoxin complex.

##### 3.3.1.10 SNAP CAR

The SNAP CAR platform uses a modified human O-6-methylguanine-DNA methyltransferase self-labeling enzyme SNAPtag that reacts with benzylguanine (BG)-conjugated antibodies, forming a covalent bond. The SNAP CAR platform is the only one that includes irreversible interactions, and the control of CAR activity is affected by the receptor turnover rate. 

##### 3.3.1.11 Target-redirected universal CAR (TRUE CAR) platform

In the TRUE CAR platform, modified exogenous antigens are loaded onto fusogenic nanoparticles to achieve *in situ* modification of the cell membrane in solid tumors, providing targets for subsequent CAR-T cell therapy.

Additional details regarding these platforms are described in **Table 2** and **Supplementary Table S1**.

#### 3.3.2 Chimeric receptor structure in the included platform

Concerning the structural characteristics of the signaling module, the vast majority of the included studies (29 out of 33) employed a second-generation CAR. These second-generation CARs featured a single costimulatory domain, which was either CD28 or 4-1BB. Only one study out of the 33 included used a first-generation CAR, which lacks a costimulatory domain. Moreover, three studies utilized a third-generation CAR with the two costimulatory domains CD28 and 4-1BB (3 out of 33 studies). The hinge domain varied widely, with CD8a-derived being the most commonly used in 48% of studies (16 out of 33), followed by CD28-derived in 36% (12 out of 33). Other variants included CD3-derived (1 out of 33), IgG4-derived (2 out of 33), and a mutant variant of the IgG4 hinge domain (2 out of 33). The transmembrane domain was primarily derived from CD28 in 55% of the studies (18 out of 33), while CD8a was the source in 42% of the studies (14 out of 33). In one study, the transmembrane domain was derived from CD3. In all included studies, the CD3ζ activation domain was utilized. The detailed structure of CAR is described in **Supplementary Table S1**.

#### 3.3.3 Soluble module characteristics in the included platform

##### 3.3.3.1 Antigen-binding domain

The choice of the antigen-binding domain varied across the studies, as it plays a crucial role in shaping the CAR-T therapy response. Some platforms, such as anti-FITC CAR, CD16 CAR, SNAP CAR, the SpyTag/SpyCatcher and the convertibleCAR platforms, are designed to allow for the conjugation of monoclonal antibodies (mAb) with the adapter (switch) molecule. Consequently, 9 of the included studies used monoclonal antibodies as the antigen-binding molecule. Notably, 5 of these studies used therapeutically approved monoclonal antibodies: Kudo et al., Ochi et al., Landgraf et al., and Tamada et al. utilized Rituximab targeting CD20, while Ruffo et al. and Minutolo et al. employed Trastuzumab targeting HER2. This approach is advantageous as it allows for the repurposing of these antibodies in the context of CAR therapy. Stock et al. focused on the mutation P329G L234A/L235A (LALA). This represents a recent advancement in the antibody field, where effector-silenced antibodies are generated by incorporating P329G and L234A/L235A (LALA) mutations into the Fc region. These modifications prevent unwanted immune effector functions by disrupting the antibody’s interaction with Fcγ receptors (FcγR) (27). Kuo et al. used meditope technology to design the soluble module in their platform, known as meditope-enabled mAb (memAb). They grafted a cyclic, 12 amino acid peptide onto the human anti-HER2 mAb at a site between the light and heavy chains, naming it a meditope due to its position (28).

The majority of the included studies (13 out of 33) employed single-chain variable fragments (scFv) derived from monoclonal antibodies specific to the target antigen. Conversely, 4 studies utilized the F_AB_ format as the antigen-binding domain, which comprises the heavy chain variable domain, heavy chain constant domain CH1, and light chain variable and constant domains (VH-CH1-VL-CL). Ma et al., using the anti-FITC CAR platform, preferred the F_AB_ format over monoclonal antibodies due to its short half-life, allowing better control of CAR-T activity (29). One study by He et al. utilized nanobodies (nb) to target antigens (30). Nanobodies are the variable domains of heavy-chain-only antibodies (HCAbs) found in camelids and sharks. These HCAbs are heavy-chain homodimers lacking a light chain, which reduces the size of antigen-binding part to one domain (31).

Three studies utilized receptor-ligand interactions to control CAR activity. Specifically, folate was used to target the folate receptor (44, 50), 2-[3-(1,3-dicarboxypropyl)ureido] pentanedioic acid (DUPA) to target the prostate-specific membrane antigen (43, 44), and acetazolamide (AZA), an inhibitor of carbonic anhydrase IX (44). As the target antigens’ expression is not always tumor-restricted, using ligands as antigen-targeting domain may present a risk of on-target/off-tumor toxicity.

Stepanov et al. utilized Designed Ankyrin Repeat Proteins (DARPins) G3 and 9.29 to target HER2. The G3 DARPin binds to the HER2 membrane-proximal domain IV, while the 9.29 DARPin interacts with the membrane-distal subdomain I (32). Typically, ankyrin repeat proteins are composed of tightly packed repeats, usually 33 amino acid residues each. Each repeat forms a structural unit consisting of a β-turn followed by two antiparallel α-helices, and up to 29 consecutive repeats can be found within a single protein (33).

Sun et al. employed a unique strategy for antigen targeting by loading nanoparticles with an antigen peptide from EGFR vIII (referred to as EvIII). This peptide is effectively targeted by CAR-T cells and is convenient for synthesis and modification. The resulting particles, termed fusogenic antigen-loaded nanoparticles (F-AgNPs), were used for *in situ* antigen modification (34). This approach aims to address the scarcity and heterogeneity of suitable target antigens commonly found in solid tumor.

##### 3.3.3.2 Pharmacokinetic properties of the soluble module

The presence of the soluble module in the bloodstream and within the tumor microenvironment plays a crucial role in modulating the activity of modular CAR-T cells. It provides a safety mechanism to mitigate CAR-related toxicities and prevent CAR-T exhaustion. Among the included studies, 11 studies reported pharmacokinetic assessments of the soluble module, either within the same xenograft model utilized for efficacy evaluations or in secondary models. Cartellieri et al. and Landgraf et al. specifically reported on serum levels of the soluble module post-administration. Cartellieri et al. observed that following intravenous injection of 250 ng/g of the soluble module, blood concentrations peaked at 480 ± 156.78 ng/mL after 1 hour, diminishing almost entirely within 600 minutes (26). Meanwhile, for intraperitoneal injection, the maximum blood concentration reached 400 ± 186.48 ng/mL after 120 minutes. Landgraf et al. investigated the serum concentrations of the soluble module Rit-S3 in a subcutaneous xenograft model following the co-administration of CAR-T cells with the soluble module. Remarkably, the serum levels of Rit-S3 remained stable throughout the study period, maintaining consistency until day 21, where levels peaked at approximately 600 ng/mL (3.2 nM), suggesting a robust presence of armed peripheral CAR cells. However, by day 45, no measurable levels of Rit-S3 were detected (35).

The studies by Bejestani et al., Cartellieri et al., Loff et al., Meyer et al., Ochi et al., Peng et al., Ruffo et al., Su et al., and Sun et al. investigated various aspects of pharmacokinetics and biodistribution in preclinical models. Bejestani et al. examined the tumor biodistribution and plasma pharmacokinetics of TM-PSCA, revealing rapid clearance post-intravenous and intraperitoneal injections (36). Cartellieri et al. evaluated the pharmacokinetics of anti-CD123/CD33 TM, demonstrating rapid clearance from peripheral blood post-intravenous injection with a half-life of approximately 1 hour (26). Loff et al. studied the pharmacokinetic properties of TM123, showing rapid clearance from the peripheral blood (a plasma half-life of 27 minutes) and bone marrow infiltration (20). Meyer et al. modified TM123 to increase its plasma half-life by increasing its hydrodynamic volume via fusion with sc4-1BBL, resulting in TM123-4-1BBL with a larger size (93.5 kDa), significantly extending its terminal plasma half-life to 5.8 hours post-intravenous injection and 8.6 hours post-intraperitoneal injection (37). Ochi et al. explored the antibody-dependent cell-mediated cytotoxicity activity (ADCC) of cCD16ζ-T cells against tumor cell lines *in vitro*, demonstrating dose-dependent responses, with cCD16ζ-T cells exhibiting ADCC activity at antibody doses lower than the pharmacological range (38). Peng et al. conducted pharmacokinetic profiling of Fab-N switches, determining their half-lives in CD-1 mice, ranging from 8.89 to 9.40 hours (39). Ruffo et al. developed a mathematical model to study universal adapter receptor signaling (23). Su et al. evaluated the PK properties of CTE-19.20 proteins in Balb/c and NSG mice, where CTE-19.20-His displayed a similar half-life in both strains, while CTE-19.20-RG exhibited a shorter-than-expected half-life in Balb/c mice due to albumin-mediated recirculation via FcRN binding (40). Sun et al. explored the biodistribution and tumor targeting of F-AgNPs, demonstrating their accumulation at tumor sites, peaking at 24 hours and gradually metabolizing thereafter, returning to baseline levels around 120 hours post-administration. F-AgNPs exhibited superior antigen modification efficiency compared to conventional nanoparticles (34).

Landgraf et al. investigated the pharmacokinetics of two MicAbody variants, where ULBP2-S3 is conjugated to either the light chain (LC-U2S3) or the heavy chain (HC-U2S3) of Rituximab. Both variants exhibited a β-phase similar to the parental antibody, with a sharper α-phase attributed to the retention of U2S3 binding to endogenous mouse wild-type NKG2D. The LC-U2S3 fusion demonstrated a slightly longer terminal half-life than the HC-fused MicAbody and was more effective at early time points in suppressing Raji B cell lymphoma expansion in NSG mice (35).

The majority of studies (82%, 27/33) featured proof-of-concept *in vitro* and/or *in vivo* experiments demonstrating that T cells expressing the universal receptor exhibited no baseline activity in the absence of the soluble module. However, four studies lacked experimental evidence regarding the absence of baseline activity, often due to the presence of preceding articles providing such information. It is worth noting that two studies used T cells that co-express the universal CAR and the soluble module; thus, there were no reports of baseline activity (41, 42). In all the included studies, the authors demonstrated that the activity of modular CAR T cells is dependent on the presence of the soluble module in a dose-dependent manner.

#### 3.3.4 Characteristics of the experimental setup *in vivo*

In the majority of cases (88%, 29/33), the intervention followed a specific sequence, with the tumor being injected first and allowed to establish itself, followed by the administration of CAR T-cells, either with or without the soluble module, and repeated doses of the soluble module and/or CAR T-cells as part of the treatment regimen. However, in two studies, the exact time of CAR administration in days after tumor establishment was not reported (43, 44). In the other two studies, CAR T-cells were administered prior to tumor establishment to create a low-burden tumor model and allow for the CAR T-cell engraftment of the peripheral blood (26, 36). Two other studies co-administered tumor cells with CAR-T cells (45, 46). Additionally, one study included a co-intervention in which all mice received intraperitoneal injections of 1,000–2,000 IU of IL-2 twice a week for 4 weeks (47).

*In vivo* antitumor activity against xenografts is a critical factor in determining the clinical development potential of specific CAR designs. To obtain reliable readouts that mimic clinical settings, xenografts are often treated with suboptimal doses of CAR T cells. In the studies reviewed, the CAR-T doses ranged between 1 to 40 million cells, with CAR-T cells typically administered once. The soluble module was administered multiple times in all studies except for those by Saleh et al., Landgraf, and Ochi, where it was given once alongside CAR-T cells, and Ambrose et al. and Rennert et al., where it was secreted by the CAR-T cells. **Supplementary Table 1** contains more comprehensive details regarding the intervention employed in each study.

### 3.4 Risk of bias in studies

The evaluation of study bias was conducted in accordance with the SYRCLE risk of bias tool tailored for preclinical animal investigations (13). Our analysis revealed that 45.45% of the examined studies exhibited a high risk of bias concerning sequence generation. This was partly due to the use of simple randomization methods without precisely defining the random component involved in sequence generation when mice were randomized prior to the experiments. Approximately 30.30% of the studies reported the baseline characteristics of the animals that may influence the outcomes across groups (e.g., body weight, tumor volume, or burden at the start of the experiment). Allocation concealment information was notably absent in most cases, although two studies, conducted by He et al. and Sun et al., stated that investigators were not blinded to allocation during both experimentation and outcome assessment (30, 34). All studies had an unclear risk of bias concerning the random housing of animals during experiments as well as the random outcome assessment. Notably, one study by Stock et al. (2022) reported conducting all experiments in a randomized fashion without specifying the randomization component (48). The majority of studies did not provide information related to detection bias (72.73%). Nevertheless, most studies exhibited a low risk of bias concerning incomplete outcome data (63.64%) and selective outcome reporting (93.94%). Furthermore, an additional source of bias observed across studies pertained to design-specific considerations. Specifically, in some instances, one component of the intervention, namely CAR-T cells, was introduced before the administration of tumor cells, raising questions about the potential influence of CAR-T cell presence on tumor engraftment. A comprehensive overview of the risk of bias assessments for all included studies can be found in **Figure 3B**.

### 3.5 Evaluation of modular CAR-T platforms across included studies

#### 3.5.1 Tumor burden

To evaluate CAR-T *in vivo*, there are critical parameters to be assessed and are related to the wide range of CAR-T-functions after encountering the target tumors in an organism. The primary measure of CAR-T cell functionality is their killing activity, which is assessed through changes in tumor burden or volume in xenograft models compared to negative or positive control groups. Although this measure cannot precisely predict the performance of CAR-T therapy in clinical context, it provides an estimation of the cell product’s ability to induce remission.

Out of the 33 studies analyzed, 19 reported measurements of tumor burden in treated animals, while 15 reported tumor volume measurements. However, only 10 studies for tumor burden and 11 for tumor volume were eligible for statistical synthesis. Exclusion criteria were applied due to missing reports of the central tendency measure (mean or median) and its error, absence of standard deviation or error, or normalization of outcome measures to unreported baseline values.

All of the included ten studies examined the impact of tumor burden on the efficacy of anti-tumor CAR-T therapy compared to various negative control cohorts, which included 1) untreated mice; 2) mice treated with only the switchable (soluble) module without CAR-T cells; 3) mice treated with only CAR-T cells without the switchable (soluble) module; and 4) mice treated with non-transduced T cells. Six of these studies also included a comparison of CAR-T efficacy with conventional CAR-T cells targeting the same antigen as a positive control. The negative control cohorts were combined, and two separate forest plots were utilized to represent the relevant efficacy of the included CAR-T platforms in eliminating the target tumor. The first plot compared the experimental group with the combined negative control group, while the second plot compared the experimental group to the positive control group. A total of 234 mice were included in these studies. One study by Peng et al. contributed data from two different cell lines for xenograft model establishment; accordingly, we added numerical identifiers to the study ID, as follows: 1) JeKo-1 mantle cell lymphoma cell line; and 2) HT-29 colorectal adenocarcinoma cell line (39).

**Figure 4:**
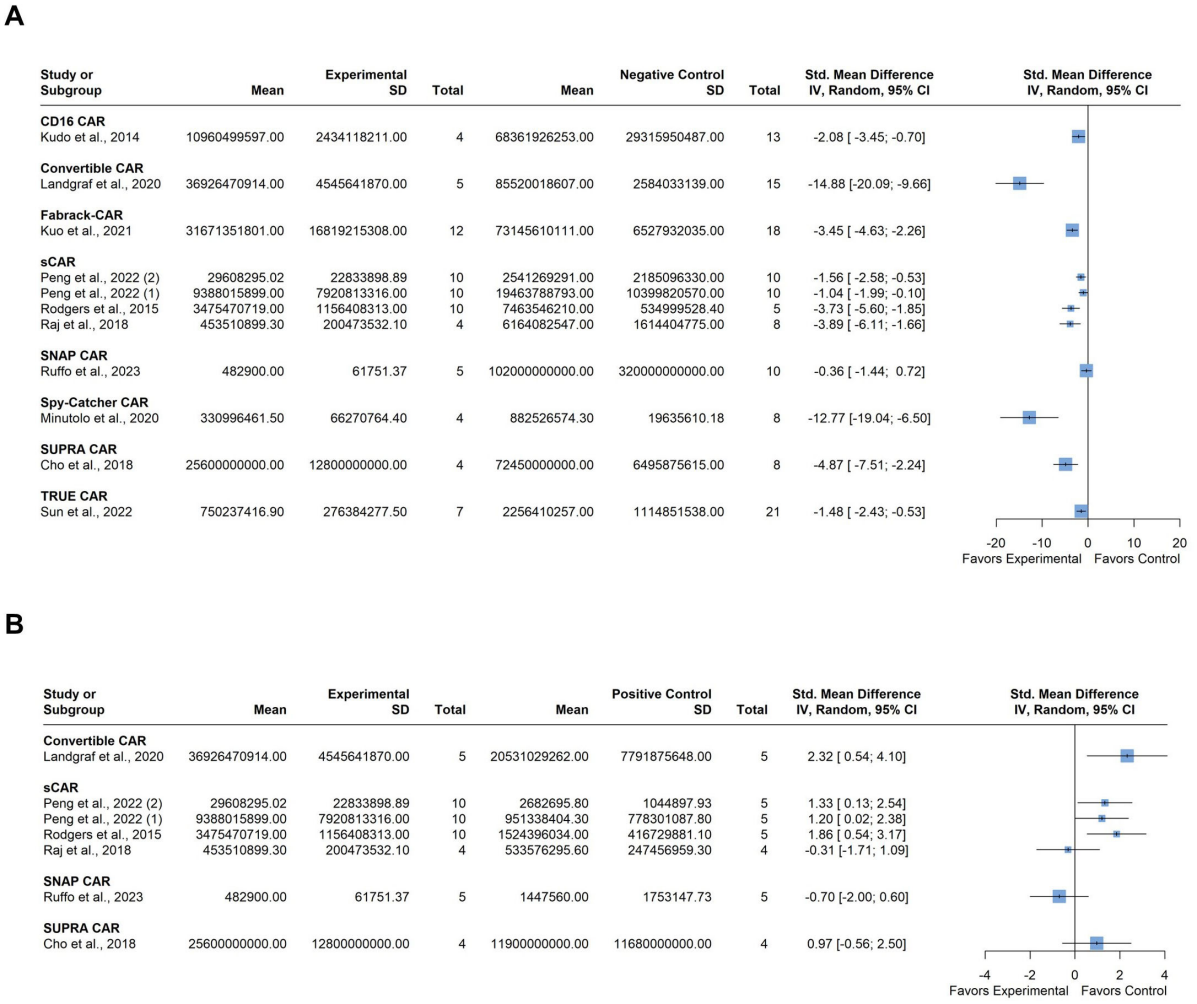
Summary effect sizes of tumor burden. **(A)** compared to the combined negative and **(B)** positive control groups. The studies are grouped according to the modular CAR-T platform used; the names of the platforms are shown in bold above the studies in which they were employed. SD, standard deviation; CI, confidence interval.

Individual studies demonstrated varying degrees of tumor burden reduction when employing modular CAR systems, each influenced by specific CAR architectures, cancer types, and experimental conditions (**Figure 4A**). Substantial between-study heterogeneity was observed, with an *I^2^* value of 84.8% (95% CI: 74.4; 90.9%). Graphic Display of Heterogeneity (GOSH) plots identified three influential outliers: 35, 47; and 54. After excluding these outliers, there was still notable heterogeneity the *I^2^* value decreased to 76.6% (95% CI: 53.4; 88.3%), likely due to the use of different modular CAR-T platforms across studies. However, the overall trend suggests a potential benefit of the experimental modular CAR-T platforms in reducing tumor burden.

Comparison with the positive control group indicated varied results among individual studies regarding tumor burden. (**Figure 4B**). For instance, some studies showed a slight tendency towards greater tumor burden in the experimental group compared to the control, but this difference was not significant. Heterogeneity in this analysis was moderate, with an *I^2^* value of 56.8% (95% CI: 0.0%; 81.4%). No influential outliers were identified in the GOSH plots despite the diverse nature of the CAR-T platforms used.

#### 3.5.2 Tumor volume

Eleven studies examined tumor volume as the primary determinant of anti-tumor CAR-T activity, comparing it to negative control cohorts. Two of these studies also included comparisons with conventional CAR-T cells targeting the same antigen as a positive control. Negative control cohorts were consolidated, and forest plots were utilized to represent the relevant efficacy of the included CAR-T platforms in eliminating the target tumor comparing either to the combined negative control group or the positive control. A total of 361 mice were involved in these studies. Four studies were included multiple times: Bejestani et al. contributed twice due to the utilization of two distinct tumor burden xenograft models, varying in CAR-T administration timing (36). The identifiers 1) denote a low-burden model with CAR-T cells administered four weeks before tumor establishment, while 2) represent a high-burden model with CAR-T cells administered four weeks after tumor establishment. Karches et al. were included three times as they employed three different cell lines for xenograft model establishment: 1) Suit-2-MSLN, 2) MIA-PaCa-MSLN, and 3) MIA MSTO-MSLN (49). Lu et al. used two different cell lines for tumor establishment, so they were included twice, as follows: 1) MDA-MB-231 and 2) Hos-FRa (50). Lastly, Lee et al. (2018) (44) utilized the MDA-MB-23 cell line, which naturally expresses FRa, and the same cell line engineered to express PSMA, or carbonic anhydrase (CA IX) (44). The identifiers (1) (2) (3), were assigned to these models, respectively.

**Figure 5:**
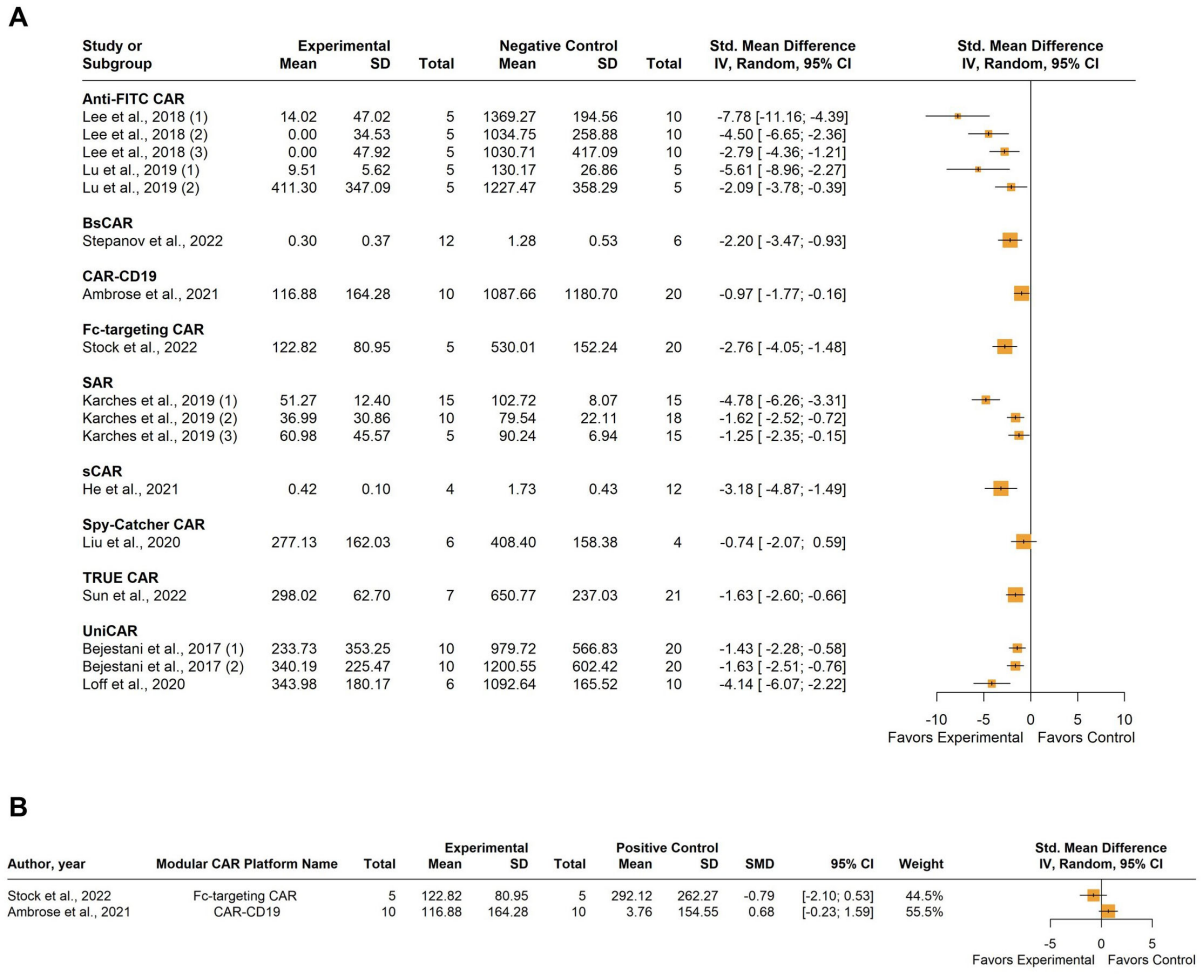
Summary effect sizes of tumor volume. **(A)** compared to the combined negative and **(B)** positive control groups. The studies are grouped according to the modular CAR-T platform used; the names of the platforms are shown in bold above the studies in which they were employed. SD, standard deviation; CI, confidence interval.

As expected, individual studies demonstrated varying degrees of tumor volume reduction when employing modular CAR systems. However, the overall tendency shows a reduction in the tumor volume after treatment compared to the negative control. The between-study heterogeneity variance was estimated at *I^2^* = 72.7% (95% CI: 55.8%; 83.2%) (**Figure 5A**). Graphic Display of Heterogeneity (GOSH) plots identified three influential outliers: 41; (1, 49); and (1, 44). The *I^2^* value dropped to 52.6% (95% CI: 13.0%; 74.2%) after outliers’ exclusion. This moderate heterogeneity probably stems from the use of different modular CAR-T platforms. In the two studies using positive control for comparison, no significant difference in tumor volume was observed (**Figure 5B**).

#### 3.5.3 Peripheral blood T cell quantification and phenotype

It is essential to comprehend the dynamic changes in the quantity of CAR T cells and their phenotype, which indicates the developmental and functional stages of CAR T cells. The phenotype of CAR T cells is defined by the expression of certain surface markers that occur during their differentiation, activation, and memory formation. Typically, CAR T cells with a memory-like phenotype, characterized by the expression of surface markers CD62L, CCR7, CD45RA, and CD45RO, exhibit superior efficacy. Analyzing these markers on CAR T cells, alongside other functional tests, would provide a thorough understanding of the underlying biology of how CAR T cells maintain or lose their antitumor function.

Six studies examined the CD3^+^ CAR^+^ T-cell count in peripheral blood following T-cell injection, with He et al. (30), Lu et al. (50), and Ma et al. (29) reporting events per μL of blood, while Liu et al. (51) and Meyer et al. (37) reported it as a percentage of total lymphocytes. The duration of observation varied across studies, ranging from 10 to 54 days post-CAR-T cell administration. Only Liu et al. and Lu et al. assessed the significance of the observed differences between the experimental and control groups. The former found no significant difference between the experimental groups and the conventional CAR-T control group. The latter found a significant difference (*p*<0.05) between the experimental group and the CAR-T-only group, with a tenfold higher prevalence of CAR-T cells in the blood of the experimental groups in both the THP1-FRβ and MDA-MB-231 models.

Ruffo et al. (23) reported the percentage of various CAR-T cell phenotypes in peripheral blood after 7 days of CAR-T administration, including T-central memory (TCM) CD62L^+^CD45RA^-^, T-effector memory (TEM) CD62L^-^CD45RA^-^, terminal effector memory T cells (TEMRA) CD62L^-^CD45RA^+^, and T-stem cell memory (TSCM) CD62L^+^CD45RA^+^, without performing statistical tests for comparison with conventional CAR-T control because the results were reported for one sample per each group. Stock et al. (48) included the following phenotypes: naïve-like T (TN) cells as CD45RA^+^ CCR7^+^, central memory-like T (TCM) cells as CD45RA^-^ CCR7^+^, effector memory-like T (TEM) cells as CD45RA^-^ CCR7^-^, and effector-like T (Teff) cells as CD45RA^+^ CCR7^-^. Compared to the conventional CAR-T control, significant differences were observed for the TN (p<0.05), TCM (*p*<0.001), and TEM (*p*<0.0001) populations, while no significant difference was observed for Teff. However, after 10 days of treatment, CAR-T cells in the experimental group predominantly exhibited the effector memory-like T (TEM) phenotype.

Rodgers et al. investigated the impact of different doses of the soluble module (0.05, 0.5, or 2.5 mg/kg) on the proportions of various memory phenotypes (TCM, TEM, TEMRA, TSCM) compared to conventional CAR T cells. They examined the expression of CD45RA and CD62-L on CD4+ and CD8+ T cells in peripheral blood after 21 days (5 days following the final dose). The group receiving 2.5 mg/kg exhibited significant expansion of the CD45RA+CD62L− terminal effector memory expressing CD45RA (TEMRA) compartment in both CD4+ and CD8+ T cells, compared to the lower-dose groups. Conversely, the lower-dose groups (0.05 and 0.5 mg/kg) had significantly larger populations of CD45RA−CD62L+ central memory cells, which is hypothesized to result from lower levels of stimulation during the initial dosing period. This finding highlights a key advantage of this platform: the dosing of the soluble module can influence the CAR-T memory phenotype *in vivo* (24).

A detailed representation of this outcome is represented in **Supplementary Table 2**.

#### 3.5.4 Median survival

All 18 studies included in the analysis reported survival rates in treated animals compared to negative control groups, with 5 studies also using conventional CAR-T as a positive control for comparison. Therefore, two separate forest plots were constructed to show the median survival relative to the combined negative group or positive group. A total of 518 mice were involved in the statistical synthesis. Seven studies were included multiple times: Bejestani et al. contributed twice due to using two distinct tumor burden xenograft models with varying CAR-T administration timing (36). The identifier (1) denotes a low-burden model with CAR-T cells administered four weeks before tumor establishment, while (2) represents a high-burden model with CAR-T cells administered four weeks after tumor establishment. Benmebarek et al. used (1) MV4-11-LUC-GFP and (2) THP-1-LUC-GFP cell lines to establish two xenograft models (52). Kuo et al. targeted two different tumor antigens (1): EGFR/HER3, and (2) CDH6 (28). Loff et al. used two different xenograft models (1): cell-line-derived and (2) patient-derived (20). Peng et al. contributed data from two different cell lines for xenograft model establishment (1): JeKo-1 mantle cell lymphoma cell line; and (2) HT-29 colorectal adenocarcinoma cell line with R12 N Fab soluble model; and (3) with 324 N Fab soluble model (39). Rennet et al. used two cell lines for xenograft model establishment (1): U937 and (2) PL21 (42). Sun et al. also used two cell lines (1): MKN45 and (2) MGC803 (34).

**Figure 6:**
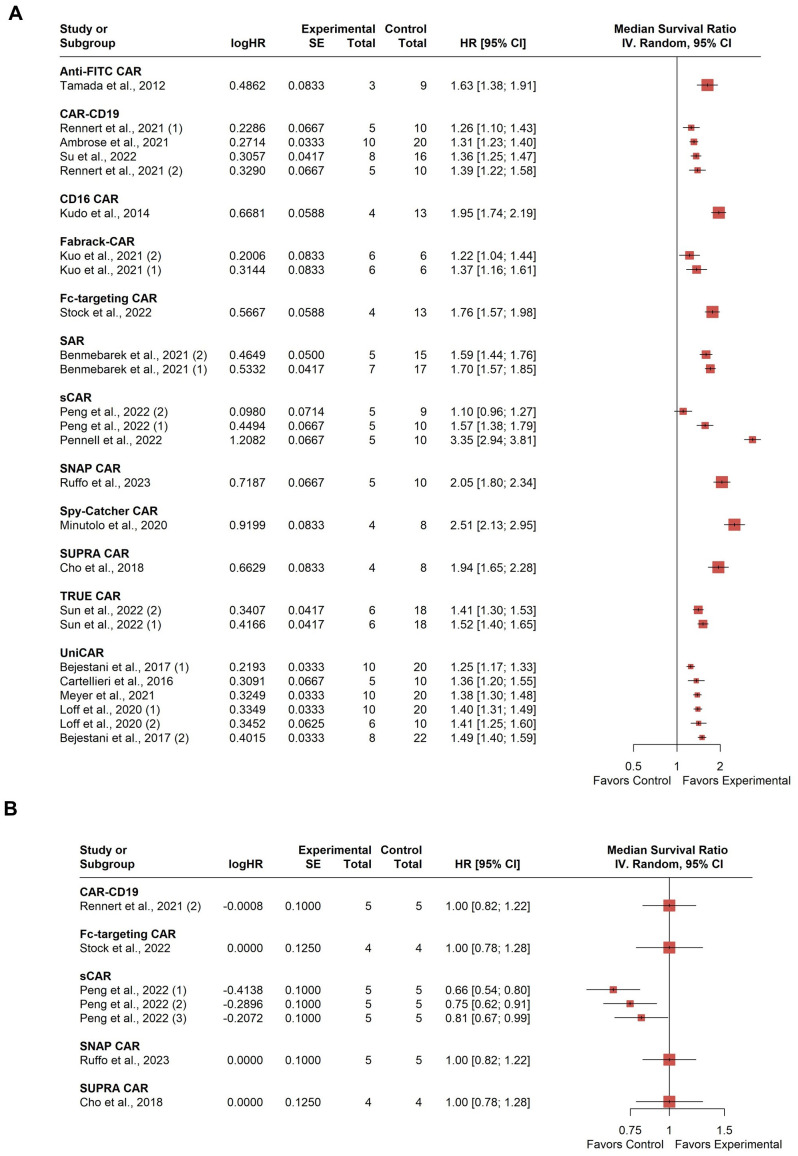
Summary effect sizes of median survival. **(A)** compared to the combined negative and **(B)** positive control groups. The studies are grouped according to the modular CAR-T platform used; the names of the platforms are shown in bold above the studies in which they were employed. SD, standard deviation; CI, confidence interval.

The median survival varied among studies with a clear tendency towards a higher survival probability compared to the combined negative group at the study endpoint (**Figure 6A**). However, substantial between-study heterogeneity was observed, with an *I^2^* value of 93.3% (95% CI: 91.2%; 94.8%). Graphic Display of Heterogeneity (GOSH) plots identified three influential outliers (1, 26, 28, 41). Excluding these outliers, the *I^2^* value remained high at 93.8% (95% CI: 91.8%; 95.3%), indicating considerable heterogeneity presumably related to the use of different modular CAR-T platforms across studies, as well as the different target antigens and experimental setups. Comparing to the positive control group, there is no significant difference in survival rate, with a minor tendency toward a lower survival rate (**Figure 6B**). Heterogeneity in this analysis was moderate, with an *I^2^* value of 63.8% (95% CI: 18.2%; 84.0%). No influential outliers were identified in the GOSH plots.

#### 3.5.5 Body weight

Body weight was assessed either in grams or as a percentage change in body weight at the study endpoint. Eight studies provided data on this measure, and a standardized mean difference was computed to accommodate the varied measurement scales [citations]. The findings indicated either a decrease in body weight or no change compared to the combined negative control group. This trend aligns with the reduction in tumor burden and/or tumor volume observed in the experimental group (**Supplementary Figure 1A**).

#### 3.5.6 Human cytokines in the peripheral blood

Understanding the cytokine profile of CAR T cells is crucial for determining efficacy and toxicity. Certain cytokines, such as IL-2, IL-15, IFN-γ, and TNF-α, can promote CAR T cell expansion and killing, activating other immune populations. Others, like IL-6, IL-10, and TGF-β, can inhibit antitumor immune function. Human cytokine levels in peripheral blood were measured in picograms per milliliter (pg/mL), with data reported in 7 out of 33 studies. Among these, 3 studies compared cytokine levels to a conventional CAR-T against the target antigen as a positive control. All the assessed cytokines were pro-inflammatory, including IFN-γ, TNF, IL-2, IL-18, and IL-6. IFN-γ was reported in all seven studies, TNF in 4 out of 7, IL-2 in 3 out of 7, and IL-18 and IL-6 were each reported in one study. Standardized mean differences were calculated, considering either a positive control (**Supplementary Figure 1B**) or a combined negative control (**Supplementary Figure 1C**). Notably, cytokine expression was generally higher in the experimental group compared to the negative control group, while expression levels were lower or showed no significant change compared to the positive control group. In the study by Liu et al. cytokine production was noticeably lower than conventional CAR-T cells (51).

#### 3.5.7 Percent tumor-free

The percentage of tumor-free mice is another parameter to assess the ability of the CAR-T platforms to eliminate tumors. This outcome measure was calculated as the proportion of mice without tumors relative to the initial number of animals at the start of the experiment. It was assessed in only two studies: one using anti-FITC CAR targeting EGFR with the soluble module FITC-cetuximab (Ctx), and another using SpyCatcher CAR-T targeting hGPC3 with anti-hGPC3 scFv-SpyTag (51, 53). Both studies showed that the mice treated with modular CAR-T remained tumor-free until the end of the experiment. Tamada et al. found that anti-FITC CAR with FITC-Ctx effectively prevented tumor formation until day 26 in the experimental group, while control groups had tumors by day 15 (53). In Liu et al.’s study, the highest concentration of anti-hGPC3 scFv-SpyTag kept mice tumor-free for 50 days, whereas lower concentrations and control groups did not (51).

#### 3.5.8 Metastases formation

Five studies explored modular CAR-T therapy efficacy against tumor metastasis in different experimental models. Lu et al. utilized a xenograft model of acute myeloid leukemia (AML) to investigate metastasis, where THP1-FRβ tumor cells were administered intravenously, leading to widespread dissemination of the tumor cells, forming both liver and non-liver metastatic lesions, most of which localized to the mouse ovary. Treatment with Spy-Catcher CAR cells plus EC17 effectively controlled liver tumor metastases compared to control groups (54). In contrast, Meyer et al. found that UniCAR-T-treated animals developed extramedullary AML disease. The treated animals exhibited numerous subcutaneous and organ metastases, emphasizing the aggressive nature of the disease. All studied metastases had a high CD33^+^ AML chimerism and CD123 positivity (37).

Ochi et al. observed prolonged survival in mice treated with cCD16ζ-T cells and rituximab, with significant suppression of tumor growth in the liver, spleen, lung/heart, and uterus/ovary/fallopian tube compared to control groups (38). Raj et al. used switchable CAR-T cells targeting HER2 in a patient-derived xenograft model of stage IV advanced pancreatic ductal adenocarcinoma (PDAC) to simulate the significant liver and lung metastases seen at this stage. Throughout the experiment, the mice treated with sCAR-T cells and HER2-specific switches remained tumor-free, as did mice treated with conventional HER2 CAR-T, demonstrating that the sCAR platform is promising against aggressive and diffuse tumors derived from patients with advanced PDAC (21).

Lastly, Sun et al. mimicked clinical scenarios by establishing a disseminated peritoneal tumor model. They found that combined administration of the soluble module F-AgNPs and TRUE CAR-T cells effectively controlled tumor growth and cleared metastatic regions (34).

### 3.6 Other platforms that were not included

One shortcoming of this review is that the selection criteria excluded some valuable and newly established platforms worth mentioning. Therefore, we briefly describe these platforms here, highlighting their *in vivo* activity where it has been examined.

#### 3.6.1 Biotin-binding immunoreceptor

The earliest platform was developed by Powell Jr. lab in the University of Pennsylvania and it is based on the high-affinity interaction between avidin and its natural binding partner, biotin. The chimeric receptor, called biotin-binding immune receptor (BBIR), consists of the second-generation CAR structure with avidin as the ectodomain so it specifically recognizes biotinylated antibodies. Interestingly, CAR T-cells were only activated when biotinylated antibodies bound to their target. Free biotin, on the other hand, can bind to the receptor and render CAR T-cells inactive (55). This strategy has been proven effective in the elimination of tumors expressing EpCAM and CD20 both *in vitro* and *in vivo* (55, 56). However, Avidin is considered xenogeneic, thus there are some concerns about its immunogenicity. The authors claimed that the presence of a preconditioned environment (lymphocyte depletion) can reduce the risk of developing inhibitory immunogenicity (55).

#### 3.6.2 Latching Orthogonal Cage–Key pRotein system

One of the innovative universal CAR platforms is called multiple component-logically gated CAR or CO-LOCKER CAR. It uses the Latching Orthogonal Cage–Key pRotein (LOCKR) switch which consists of a structural “Cage” protein that uses a “Latch” domain to sequester a functional peptide in an inactive conformation until binding of a separate “Key” protein induces a conformational change that permits binding to an “Effector” protein. This allows for the logical gating of CAR activity (AND, OR, NOT gates). The platform was only tested *in vitro* as a “proof-of-principle”. *In vivo* studies, however, have not been conducted yet (57).

#### 3.6.3 Adapter CAR system

The AdCAR-T platform employs a two-component signal transduction system based on a split recognition/activation design, where labeled monoclonal antibodies transmit antigen recognition into T-cell activation via an anti-label CAR. The soluble module in this system is generated through biotinylation using specific linker chemistry, resulting in a molecule comprising an antigen-binding moiety, a linker moiety, and a label moiety (biotin). The AdCAR is based on the unique characteristics of the monoclonal antibody mBio3, which binds to biotin in the context of a specific linker, referred to as a Linker-Label Epitope (LLE). The authors tested the *in vivo* activity of this platform using a rapidly progressive xenograft model of Burkitt’s lymphoma (Raji cell line) and utilized the therapeutic mAb Rituximab as the antigen-binding domain to demonstrate the potential for direct clinical translation of the AdCAR-T technology. They did not observe signs of GvHD in the mouse models. Tumor burden was assessed by *in vivo* bioluminescence imaging (BLI). Remarkably, AdCAR-T in combination with LLE-rituximab completely eradicated disseminated lymphoma, proving as efficient as conventional CD20-CAR-T, although with slightly delayed kinetics. Mice remained in complete remission, as demonstrated by BLI and flow cytometry of bone marrow, even after LLE-rituximab administration was terminated. In contrast, neither AdCAR-T nor LLE-rituximab alone had a significant effect on tumor burden (58).

#### 3.6.4 ARC-SparX platform

The ARC-SparX platform combines a genetically modified T cell expressing an antigen-receptor complex (ARC-T) with a soluble protein antigen-receptor X-linker (SparX) that links the ARC-T to tumor cells. ARC-T cells feature a 73 amino acid synthetic protein D-domain in their extracellular domain, specifically binding to the TAG domain within the SparX adapter, which includes the third domain of human alpha-fetoprotein (AFP domain III) due to its stability and human origin. The SparX adapter also utilizes D-domains engineered to bind various tumor antigens. The platform’s selectivity ensures ARC-T cell activation only in the presence of SparX and antigen-expressing tumor cells.

A key advantage of the ARC-SparX platform is the controllability of ARC-T cells through SparX protein administration. *In vivo* studies on NALM6-BCMA-bearing NSG mice revealed that BCMA SparX proteins bound to tumor cells were detectable up to 8 hours post-injection. Cytokine levels in serum, such as IFN-γ, peaked at 8 hours and dropped to baseline within 48 hours after BCMA SparX administration, demonstrating intermittent cytokine activation. This pattern differed from the traditional BCMA-CAR, where cytokine levels steadily increased.

Comparative studies using a xenograft model showed that ARC-T cells with daily doses of bivalent BCMA SparX cleared tumors as effectively and rapidly as BCMA-CAR. Both cell types expanded *in vivo* to a peak on day 7, with BCMA-CAR cells expanding more extensively. At peak expansion, both cell types displayed similar effector phenotypes and increased effector memory (TEM) and effector T cells (TEMRA), while reducing stem-cell memory phenotype T cells (59).

#### 3.6.5 Conduit CAR

The main advantage of the conduit CAR platform is that it does not require the introduction of any novel engineered antigen on T cells, but rather utilizes an existing feature present in most clinical CAR T-cell therapies, the flexible ScFv linker. The soluble module is a bispecific antibody (BsAb) that targets the (GGGGS)n or (G4S)n linker found on most existing CARs and the tumor-associated antigen. Borrok et al. demonstrated that CAR T cells expressing either a germline antibody ScFv (with no known specificity) or a CD19-targeting CAR can be redirected to target prostate tumor cells via a bispecific soluble module. This technology offers clinical advantages due to its adaptability to target novel TAAs, potential toxicity control through dosage adjustment, and compatibility with existing clinical CARs. However, the study did not include *in vivo* experiments (60).

#### 3.6.6 Folate receptor (BsAb-binding immune receptor)

This platform relies on the interaction between folate and the folate receptor and combines the application of a bispecific antibody (BsAb) with T-cells genetically engineered to express a unique BsAb-binding immune receptor (BsAb-IR). The BsAb-IR consists of a portion of an extracellular folate receptor (FR; 231aa) fused to intracellular TCR and CD28 costimulatory signaling domains in tandem. It can be bound and activated by an anti-FR antibody arm of a unique BsAb that bridges the FR and tumor antigen (frBsAb). The study included a proof-of-concept *in vitro* experiment showing that tumor antigen-specific frBsAbs bind specifically to target antigens on human tumor cells. Upon co-engagement of the BsAb-IR on engineered T-cells, this binding delivers simultaneous TCR CD3 activation and CD28 costimulation signals in a target-dependent manner. This results in the selective augmentation of activation, proliferation, and antitumor activity of the BsAb-IR T-cell subset. However, no *in vivo* studies were conducted (61).

## 4 Discussion

Modular CAR platforms offer a novel solution to overcome the limitations of current CAR technologies. They present a more flexible and manageable therapeutic approach, allowing for the targeting of multiple antigens. The majority of these platforms and others have undergone *in vivo* studies on animal models. Therefore, we decided to collect the available preclinical evidence on their performance *in vivo*. We conducted systematic research of the available animal studies of the modular CAR-T platforms. Our findings indicate that these platforms outperformed negative control groups in terms of tumor elimination, survival rate, and pro-inflammatory cytokine secretion, while performing equally well as their conventional CAR-T counterparts.

Despite the fact that these platforms share a similar principle, each study’s *in vivo* experimental design was very different. Therefore, we did not limit the inclusion criteria with respect to the experimental design (CAR and soluble module administration times and frequencies, for example), in an effort to be inclusive of all these studies. Nevertheless, our analysis did not fully represent all of the modular CAR T platforms that are currently available. Because of the variety of platforms, targeted antigens, experimental designs, and xenograft models employed, there was a noticeable heterogeneity among the studies. On the other hand, heterogeneity between studies is commonly observed in systematic reviews of animal studies, and it is mostly due to the variable nature of animal studies in terms of study characteristics and design (62).

The soluble module, the controlling component that regulates CAR T cell activity, is what sets modular CAR-T platforms apart from conventional CAR-T therapy. And every study that was included supported this assertion. It is reasonable to assume that the soluble module’s biodistribution, binding affinity, and availability all affect CAR activity and, consequently, platform safety. Not every study explicitly mentioned this element; in those cases, we looked into whether earlier reports had addressed the issue. The selection of the soluble module dose range and administration frequency, however, seems to be primarily empirical, though we were unable to ascertain the rationale behind it.

When conducting animal studies, there are guidelines that should be followed in order to ensure repeatable and dependable results with the least amount of animal sacrifice. During our research, we often discovered that the animal studies were not adequately reported, particularly when it came to the methods used in the animal experiments. Some studies even failed to disclose the total number of mice used in the experiments or the reasoning behind selecting the appropriate sample size. Not all the data was provided in the article or in the supplementary materials. The lack of clarity and appropriate reporting threatens the research’s reproducibility and validity.

In conclusion, we found that the modular CAR-T platforms are generally effective and are a valuable addition to the arsenal of CAR therapy.

## Data Availability

The datasets presented in this study can be found in online repositories. The names of the repository/repositories and accession number(s) can be found below: https://github.com/afraa-create/Meta-analysis-of-modular-CAR-platforms.git.
