# Supplementary material for: Modular (universal) CAR-T platforms in vivo: a comprehensive systematic review
Source: Front Immunol. 2024 Dec 6;15:1409665. doi: 10.3389/fimmu.2024.1409665 (PMC11659234; doi:10.3389/fimmu.2024.1409665)
Supplement: Supplementary file 1 [file DataSheet1.pdf]

Supplementary Table 1. Intervention details in the included studies.

Author, year	CAR Domain Structure [a]					CAR administration times, doses [b]	Soluble module doses	Frequency of soluble module administration (No. of times)	Duration (Days)
	ECD	H	TM	costim	Act				
Ambrose et al., 2021	FMC63 anti-CD19	CD28	CD28	CD28 and 4-1BB	CD3ζ	Day +14, 1×10^6	Soluble module is secreted by CAR T-cells		16
Bejestani et al., 2017	anti-La/SS-B 5B9 scFv	CD28	CD28	CD28	CD3ζ	Day -28, 1×10^6<br>Day +28, 1×10^6	25 mg/mouse	Multiple (14 times)	98-154
Benmebarek et al., 2021	E3(EGFRvIII)	CD28	CD28	CD28	CD3ζ	Day +5, 10×10^6	2.5 μg/mouse	Multiple (19 times)	50
Cartellieri et al., 2016	anti-La/SS-B 5B9 scFv	CD28	CD28	CD28	CD3ζ	Day -28, 1×10^6	250 ng/g	Multiple (4 times)	15
Cho et al., 2018	Leu zipper	CD8a	CD28	4-1BB	CD3ζ	Day +14 (SK-BR-3 model), 35×10^6<br>Day +3-5 (Jurkat T model), 35×10^6	8 and 5 mg/kg	Multiple (8 times)	40 (SK-BR-3)<br>21 (Jurkat T)
He et al., 2021	Anti-PNE scFv	CD8a	CD8a	4-1BB	CD3ζ	Day +14, 3×10^6	0.1 and 0.5 mg/kg	Multiple (every 3-4 days)	52
Hidalgo et al., 2023	Anti-FITC scFv	CD28	CD28	4-1BB	CD3ζ	Day +1, 6×10^6	50 and 100 μg/mouse	Multiple (6 times)	20
Karches et al., 2019	E3(EGFRvIII)	CD28	CD28	CD28	CD3ζ	Day +5 (Suit-2-MSLN and MIA PaCa-MSLN models), 10×10^6<br>Day +2 (MSTO-MSLN model), 10×10^6	5, 10, 20 μg/mouse	Multiple<br>(Suit-2-MSLN, 4)<br>(MIA PaCa-MSLN, 9)<br>(MSTO-MSLN, 5)	22 (Suit-2)<br>36 (MIA)<br>23 (MSTO)
Kegler et al., 2019	anti-La/SS-B 5B9 scFv	CD28	CD28	CD28	CD3ζ	Day 0, 1×10^6	10 μg/mouse	Single (with CAR-T)	19
Kudo et al., 2014	CD16 (FCGR3A) V158	CD8a	CD8a	4-1BB	CD3ζ	Day +5-6, 10×10^6	150 μg/mouse	Multiple (4 times)	120
Kuo et al., 2021	CQFDLSTRRLQC + (PAS) linker	IgG4	CD28	CD28 or 4-1BB	CD3ζ	Day +5, 10×10^6	1.25 mg/kg	Multiple (7 times)	29
Landgraf et al., 2020	iNKG2D.YA	CD8a	CD8a	4-1BB	CD3ζ	Day +2 (Raji, IV model), 5 and 15×10^6<br>Day +12 (Raji, SC model), 7 and 35×10^6	20, 60 μg/mouse	Single (Raji, SC model)<br>Multiple (5 times)	50 (SC model)<br>30 (IV model)
Lee et al., 2018	Anti-FITC scFv	CD8a	CD8a	4-1BB	CD3ζ	Not reported, 10×10^6	5, 50, 100, 500 nM/kg	Multiple (5 nM/kg on days 1 and 2, 50 nM/kg on days 4 and 6, 100 nM/kg on days 8 and 10, and 500 nM/kg from day 12 onward)	36
Lee et al., 2019	Anti-FITC scFv	CD8a	CD8a	4-1BB	CD3ζ	Not reported, 20×10^6	500 nM/kg	Multiple (on days 1, and 2, and alternate days thereafter)	36
Liu et al., 2020	SpyCatcher	CD8a	CD28	4-1BB	CD3ζ	Day +7, 10×10^6	1,5,10 μg/mouse	Multiple (10 times)	42
Loff et al., 2020	anti-La/SS-B 5B9 scFv	CD28	CD28	CD28	CD3ζ	Day +3 (MOLM-13 model), 5×10^6<br>Day +16, 30 (PD model), 5×10^6	1, 2.5 mg/g	Multiple (20 times)	80

Lu et al., 2019	Anti-FITC scFv	CD8a	CD8a	4-1BB	CD3ζ	Day +1 (THP1-FRb model), 6×10^6 Not reported (MDA-MB-231, HOS-FRa models), 10×10^6	5, 10, 30, 50, 100, 300, 500, 1000 nM/kg	Multiple (5 times)	40
Ma et al., 2015	Anti-FITC scFv	CD8a	CD8a	4-1BB	CD3ζ	Day +7, 40×10^6	0.5, 0.05, 0.005 mg/kg	Multiple (6 times)	60
Meyer et al., 2021	anti-La/SS-B 5B9 scFv	CD28	CD28	CD28	CD3ζ	Day +3, 5×10^6	1 μg/g	Multiple (20 times)	40
Minutolo et al., 2020	SpyCatcher	CD8a	CD28	4-1BB	CD3ζ	Day +7, 10×10^6	1000 nM/mouse	Multiple (1 day after T-cell injection, followed by subsequent injections every 3 days until treatment cessation)	100
Ochi et al., 2014	CD16	CD3	CD3	NA	CD3ζ	Day +4, 24, 5×10^6	40 μg/mouse	Simultaneously with CAR T	31
Peng et al., 2022	GCN4-binding scFv 52SR4	IgG4 m	CD28	4-1BB	CD3ζ	Day +6,16 (JeKo-1 model), 5×10^6 Day +3 (HT-29 model), 5×10^6	1, 3, 10 μg/mouse	Multiple (JeKo-1, 11) (HT-29, 6)	27 (JeKo-1) 18 (HT-29)
Pennell et al., 2022	anti-PNE scFv	CD8a	CD8a	4-1BB	CD3ζ	Day +7, 3×10^6	0.5 mg/kg	Multiple (14 times)	21
Raj et al., 2018	anti-PNE scFv	CD8a	CD8a	4-1BB	CD3ζ	Day +17, not reported	0.5 mg/kg	Multiple (14 times)	159
Rennert et al., 2021	FMC63 anti-CD19	CD28	CD28	4-1BB	CD3ζ	Day +3, $2{,}5{,}10\times10^6$	Soluble module is secreted by CAR T-cells		25
Rodgers et al., 2015	anti-PNE scFv	IgG4 m	CD8a	4-1BB	CD3ζ	Day +6, 40×10^6	0.005, 0.5, 2.5 mg/kg	Multiple (10 times)	10
Ruffo et al., 2023	SNAP catcher	CD8a	CD8a	4-1BB	CD3ζ	Day +4, 20×10^6	150 μg/mouse	Multiple (Every 3 days from day 7 to day 22)	60
Saleh et al., 2023	E5B9 tag or E7B6	CD28	CD28	CD28	CD3ζ	Day 0, 1×10^6	150 μg/mouse	Single (with CAR-T)	9
Stepanov et al., 2022	Barstar-CH2-CH3	IgG4	CD28	4-1BB	CD3ζ	Day 10, 10×10^6	5, 50, 500 nM/kg	Multiple (9 times)	50
Stock et al., 2022	P329G scFv	CD8a	CD8a	CD28	CD3ζ	Day 14, 10×10^6	5, 20, 5 μg/mouse	Multiple (twice a week)	100
Su et al., 2022	FMC63 anti-CD19	CD28	CD28	CD28 and 4-1BB	CD3ζ	Day 4, 10×10^6	0.016, 0.08, 0.4, 2 mg/kg	Multiple (14 times)	43
Sun et al., 2022	anti-EGFR vIII scFv	CD8a	CD8a	4-1BB	CD3ζ	Day +9,14 (MGC803 model), 10×10^6 Day +7,12 (MKN45 model), 10×10^6	0.4, 0.8 mM/mouse	Multiple (2 times)	73 (MGC803) 54 (MKN45)
Tamada et al., 2012	Anti-FITC scFv	CD8a	CD8a	CD28 and 4-1BB	CD3ζ	Day 1, 5×10^6	25 mg/mouse	Multiple (3 times)	45

[a] ECD = Extracellular domain of CAR, H = Hinge domain of CAR, TM = Transmembrane domain of CAR, costim = Costimulatory domain and/or domains of CAR.
[b]CAR administration times are relative to tumor cells administration time, + days are days after tumor cells administration, - days are days before and 0 days means CAR T-cells administration was performed simultaneously with tumor cells administration.

Supplementary Table 2. Frequency of CAR-T and its phenotypes in peripheral blood.

Author, year	CAR name	Target	Time of measurement	Phenotype	The frequency of phenotype (mean ± SD)		*p* value
					Experimental	Control	
**He et al., 2021**	sCAR	CD13	14 days	CD3^{+} CAR^{+}	***sCAR-T + 0.1 mg/kg switch***<br>46.73±7.5 event/µl PB (n= 4)<br>***sCAR-T + 0.5 mg/kg switch***<br>130.7±43.3 event/µl PB (n= 4)	***sCAR-T***<br>15.58 ±7.45 event/µl PB (n= 4)	Not reported
**Liu et al., 2020**	Spy-Catcher CAR	GPC3	29 days	CD3^{+} CAR^{+}	***CAR-T + 1 ug scFV-spytag***<br>11.01 ± 3.95% (n= 4)<br>***CAR-T + 10 ug scFV-spytag***<br>13.1 ± 5.8 % (n= 4)	***anti-GPC3 CAR-T***<br>14.96 ± 6.68 % (n= 4)	NS
**Lu et al., 2019**	Anti-FITC CAR	FR	31 days	CD3^{+} CAR^{+}	***CAR-T + 500 nmol/kg EC17 SIW (THP1-FRb)***<br>34.55±11.86 event/µl PB (n= 5)	***CAR-T only***<br>0.38 ± 0.25 event/µl PB (n= 5)	< 0.05
			54 days	CD3^{+} CAR^{+}	***CAR-T + 500 nmol/kg EC17 SIW (MDA-MB-231)***<br>39.74 ± 63.64 event/µl PB (n= 5)	***CAR-T only***<br>0.32 ± 0.13 event/µl PB (n= 5)	< 0.05
**Ma et al., 2015**	Anti-FITC CAR	CD19	10 days	CD3^{+} CAR^{+}	***CAR-T + 0.5 mg/kg anti-CD19-FITC Ab***<br>21.45 ± 7.37 event/µl PB (n= 6)	***anti-CD19 CAR-T***<br>31.5 ±15.42 event/µl PB (n= 6)	Not reported
			18 days	CD3^{+} CAR^{+}	***CAR-T + 0.5 mg/kg anti-CD19-FITC Ab***<br>111.26 ± 26.81 event/µl PB (n= 6)	***anti-CD19 CAR-T***<br>81.77 ± 8.04 event/µl PB (n= 6)	Not reported
			27 days	CD3^{+} CAR^{+}	***CAR-T + 0.5 mg/kg anti-CD19-FITC Ab***<br>29.49 ± 40.21 event/µl PB (n= 6)	***anti-CD19 CAR-T***<br>19.44 ± 0.0 event/µl PB (n= 6)	Not reported
**Meyer et al., 2021**	UniCAR	CD123	10 days	CD3^{+} CAR^{+}	***UniCAR T + 1 µg/g TM123***<br>45.02 ± 28.7 % (n= 3)	***UniCAR T only***<br>37.46 ± 19.94 % (n= 3)	Not reported
**Ruffo et al., 2023**	SNAP CAR	CD20	7 days	CD3^{+} CAR^{+} CD4^{+} CD62L^{-} CD45RA^{-}	***SNAP-CAR T cells + switch***<br>28 % (n= 1)	***anti-CD20 CAR T cells***<br>28.2 % (n= 1)	Not reported
				CD3^{+} CAR^{+} CD4^{+} CD62L^{-} CD45RA^{+}	***SNAP-CAR T cells + switch***<br>1.43 % (n= 1)	***anti-CD20 CAR T cells***<br>0.85 % (n= 1)	Not reported
				CD3^{+} CAR^{+} CD4^{+} CD62L^{+}CD45RA^{+}	***SNAP-CAR T cells + switch***<br>7.03 % (n= 1)	***anti-CD20 CAR T cells***<br>4.86 % (n= 1)	Not reported
				CD3^{+} CAR^{+} CD4^{+} CD62L^{+}CD45RA^{-}	***SNAP-CAR T cells + switch***<br>63.6 % (n= 1)	***anti-CD20 CAR T cells***<br>66 % (n= 1)	Not reported
				CD3^{+} CAR^{+} CD8^{+}	***SNAP-CAR T cells + switch***	***anti-CD20 CAR T cells***	Not reported

				CD62L^{-} CD45RA^{-}	28.6 % (n= 1)	35.2 % (n= 1)	
				CD3^{+} CAR^{+} CD8^{+} CD62L^{-} CD45RA^{+}	***SNAP-CAR T cells + switch*** 1.64 % (n= 1)	***anti-CD20 CAR T cells*** 2.15 % (n= 1)	Not reported
				CD3^{+} CAR^{+} CD8^{+} CD62L^{+}CD45RA^{+}	***SNAP-CAR T cells + switch*** 11.4 % (n= 1)	***anti-CD20 CAR T cells*** 12.2 % (n= 1)	Not reported
				CD3^{+} CAR^{+} CD8^{+} CD62L^{+}CD45RA^{-}	***SNAP-CAR T cells + switch*** 58.4 % (n= 1)	***anti-CD20 CAR T cells*** 50.5 % (n= 1)	Not reported
**Stock et al., 2022**	Fc-targeting CAR	HER2	10 days	CD3^{+} CAR^{+} CCR7^{+}CD45RA^{+}	***P329G-CAR + 5 µg/mouse HER2 binder*** 0.0 ± 0.0 % (n= 4)	***P329G-CAR + no binder*** 7.53 ± 4.79 % (n= 4) ***anti-HER2 CAR-T*** 0.0 ± 0.0 % (n= 4)	<0.05 NS
				CD3^{+} CAR^{+} CCR7^{+}CD45RA^{-}	***P329G-CAR + 5 µg/mouse HER2 binder*** 0.0 ± 0.0 % (n= 4)	***P329G-CAR + no binder*** 16.1 ± 3.08 % (n= 4) ***anti-HER2 CAR-T*** 0.0 ± 0.0 % (n= 4)	<0.001 NS
				CD3^{+} CAR^{+} CCR7^{-} CD45RA^{-}	***P329G-CAR + 5 µg/mouse HER2 binder*** 98.29 ± 2.05 % (n= 4)	***P329G-CAR + no binder*** 69.86 ± 4.79 % (n= 4) ***anti-HER2 CAR-T*** 99.32 ± 0.5 % (n= 4)	<0.0001 NS
				CD3^{+} CAR^{+} CCR7^{-} CD45RA^{+}	***P329G-CAR + 5 µg/mouse HER2 binder*** 0.0 ± 0.0 % (n= 4)	***P329G-CAR + no binder*** 5.14 ± 4.11 % (n= 4) ***anti-HER2 CAR-T*** 0.0 ± 0.0 % (n= 4)	NS NS

SD = standard deviation, NS = non significant.

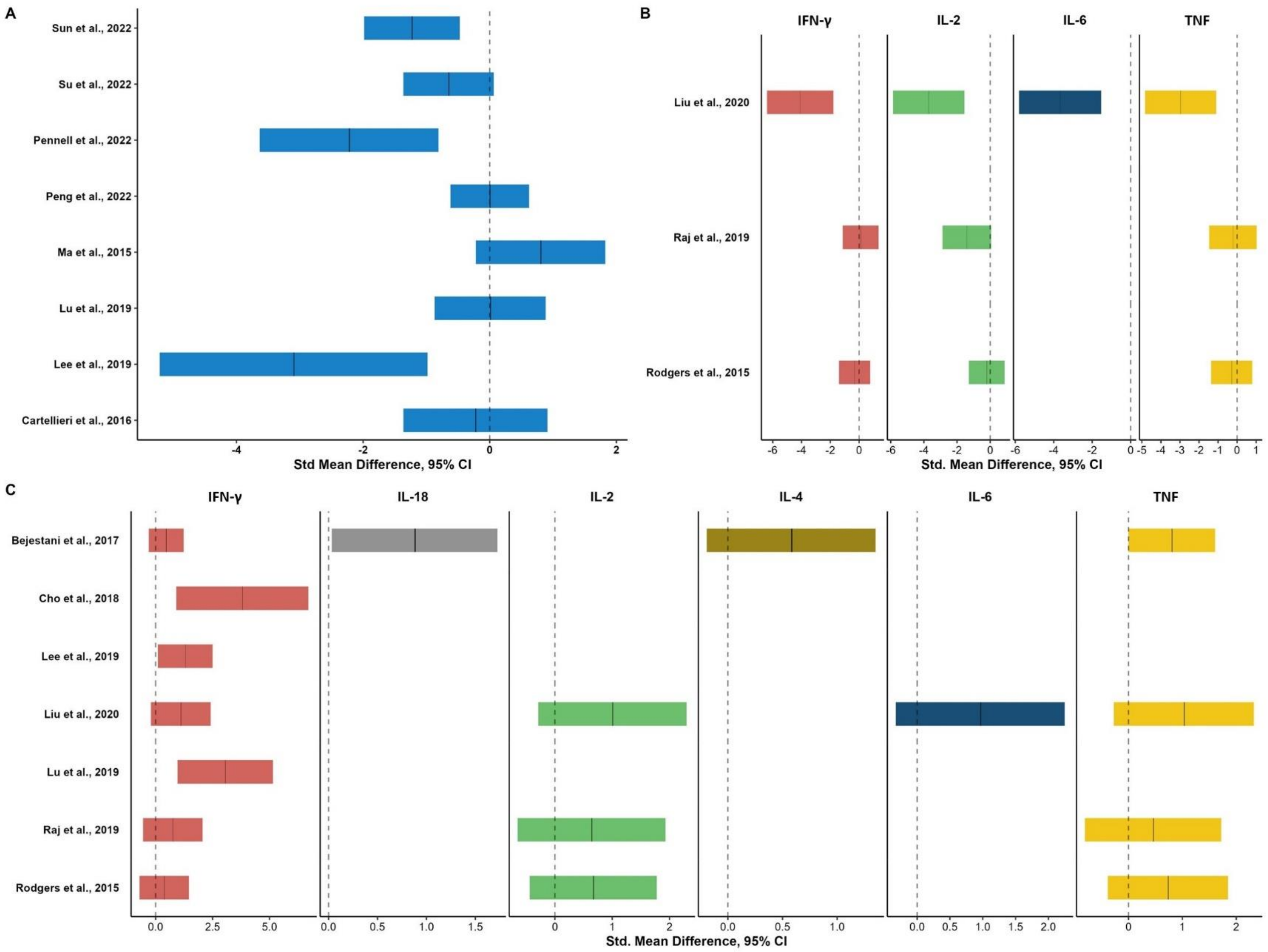

Supplementary Figure 1. Summary effect sizes of quantitative outcome measures

(A) Forest plot of the change in body weight at the endpoint of the studies shown as standardized mean difference and its 95% confidence interval between experimental group and combined negative group. Forest plots of the human cytokines detected in the bloodstream 24-48 hours after soluble module administration are shown as standardized mean difference and its 95% confidence interval between experimental group and (B) positive group or (C) combined negative control group. The dashed lines indicate the line of no effect, to the right of it effect favors the experimental group where to the left favors the control group.

Search strategy

for PUBMED

("receptors, chimeric antigen"[MeSH Terms] OR "immunotherapy, adoptive"[MeSH Terms]) AND

("universal"[Title/Abstract] OR "switchable"[Title/Abstract] OR "modular"[Title/Abstract] OR

"adaptor"[Title/Abstract] OR "split"[Title/Abstract] OR "bispecific"[Title/Abstract] OR

"UniCAR"[Title/Abstract] OR "tag"[Title/Abstract] OR "switch"[Title/Abstract]) AND ("Animal

Experimentation"[MeSH Terms] OR "models, animal"[MeSH Terms] OR "Mice"[MeSH Terms] OR "mice,

mutant strains"[MeSH Terms] OR "xenograft"[All Fields] OR "in vivo"[Title/Abstract]) AND

"Neoplasms"[MeSH Terms]

For BASE

universal chimeric antigen receptor in vivo

for Google scholar

Chimeric antigen receptor, universal, modular, split, bispecific, unicar, in vivo
